# Measuring health-related quality of life in Africa: a systematic review of validated disease-specific and generic measurement tools

**DOI:** 10.3389/fpsyg.2025.1667712

**Published:** 2026-01-07

**Authors:** AbdulMuminu Isah, Ezinwanne Jane Ugochukwu, Chinelo Ikeanyi, Obinna Onwujekwe

**Affiliations:** 1 Department of Clinical Pharmacy and Pharmacy Management, Faculty of Pharmaceutical Sciences, University of Nigeria Nsukka, Nsukka, Enugu, Nigeria; 2 Health Policy Research Group, University of Nigeria Nsukka, Nsukka, Enugu, Nigeria; 3 Department of Health Administration and Management, University of Nigeria, Nsukka, Nigeria; 4 Department of Pharmacology and Therapeutics, University of Nigeria, Nsukka, Nigeria

**Keywords:** health-related quality of life, disease-specific health-related quality of life, Africa context-specific, cultural adaptation, HRQoL

## Abstract

**Background:**

This systematic review examines the evidence on the use of health-related quality of life (HRQoL) tools for African populations and evaluates their psychometric properties, cultural adaptation, and applicability.

**Methods:**

A systematic search was conducted across PubMed, Web of Science, Scopus, and gray literature from January 2015 to January 2025. The review followed PRISMA (Preferred Reporting Items for Systematic Reviews and Meta-Analyses) and COSMIN (Consensus-based Standards for the Selection of Health Measurement Instruments) frameworks. Duplicate screening and study selection were independently performed by multiple reviewers. Eligible studies included the development, adaptation, or validation of HRQoL for African populations. The protocol was submitted for registration to the International Prospective Register of Systematic Reviews (PROSPERO) under the identification number CRD42025639055.

**Results:**

Forty studies met the inclusion criteria, with 31 (77.5%) focusing on adults and minimal attention given to the pediatric population. East Africa had the highest representation, with 17 (42.5%), while West Africa accounted for 7 (17.5%). Internal consistency (Cronbach’s alpha ≥ 0.70) was demonstrated in 33 (97.1%) out of the 34 tools. A total of 34 different HRQoL tools were identified, including 12 generic instruments. The SF-12 and WHOQOL-BREF were the most validated tools, whereas the EORTC QLQ-C30 was the most validated disease-specific tool. Cultural adaptation was a major focus, with 32 (80.0%) of the studies incorporating linguistic modifications to enhance contextual relevance. Most studies, 28 (70.0%), used cross-sectional designs. Overall, most tools demonstrated good reliability and cultural adaptability, although limitations such as small sample sizes, limited geographic coverage, and incomplete reporting of responsiveness and test–retest reliability were common.

**Conclusion:**

Significant progress has been made in developing and validating HRQoL tools for African populations. However, gaps remain, including the need for longitudinal studies, greater inclusion of children’s HRQoL assessments, and broader geographic representation. Strengthening research capacity will be pivotal in advancing culturally responsive HRQoL tools and integrating them into healthcare decision-making in Africa.

**Systematic review registration:**

https://www.crd.york.ac.uk/PROSPERO/view/CRD42025639055, Identifier: CRD42025639055.

## Introduction

Health systems that provide good-quality care aim not only to prevent and treat diseases but also to improve the wellbeing and quality of life (QoL) of patients (WHO, 2020a). QoL is a multidimensional concept that refers to an “individual’s perception of their position in life in the context of the culture and value systems in which they live and in relation to their goals, expectations, and standards” and is affected by a person’s physical health and psychological state (The Whoqol Group, 1998).

The improvement of QoL and other outcomes through proper prevention and treatment mechanisms is at the heart of clinical science and all health systems. Outcomes could be economic, clinical, or humanistic. Clinical outcomes in patient care measure the impact of the treatment on the patient, especially their QoL. Hence, the assessment of QoL should consider multidimensional aspects of physical and psychological health (Vagetti et al., 2014).

Health-related quality of life (HRQoL), which is derived from QoL, is a multidimensional concept that reflects an individual’s perception of the impact of a disease state or intervention on their physical, psychological, and social aspects of life (Tran et al., 2012; European Medicine Agency, 2006). HRQoL is essential in inpatient follow-up and monitoring, as it provides valuable feedback from the perspective of patients about a disease condition and its accompanying interventions (Mafirakureva et al., 2016).

The HRQoL measurement has emerged as an essential health outcome in clinical trials, clinical practice improvement strategies, and healthcare services research and evaluation (Varni et al., 2003). Research on HRQoL often explores patient-reported outcome measures (PROMs) that can offer valuable insights into therapeutic interventions, health strategies, and health policy development (Brazier et al., 2002; Miller et al., 2021; Conrad and Barker, 2010). This patient-centered exploration of the experience of health is particularly important, as the disjuncture between patients’ subjective experience of treatment and wellbeing and clinical improvements has been observed (Brazier et al., 2002).

HRQoL instruments are commonly grouped into generic and disease-specific measures, each serving distinct purposes in health assessment (Lipton et al., 2013). Generic instruments, such as the SF-36, SF-12, EQ-5D, and WHOQOL-BREF, assess broad domains of functioning, such as physical, emotional, and social, allowing comparisons across diseases and populations (Hand, 2016). These tools are widely used in Africa, and several validation studies report acceptable psychometric properties, particularly in internal consistency and construct validity. For example, the WHOQOL-BREF has demonstrated reliability across multiple African languages; however, cultural discrepancies have been reported regarding items related to social relationships, spirituality, and environmental context (Colbourn et al., 2012; Bowden et al., 2002; Price et al., 2020). These findings suggest that although generic tools are broadly applicable, they may overlook culturally embedded expressions of wellbeing. In contrast, disease-specific instruments—such as the EORTC QLQ-C30 for cancer, the KDQOL for kidney disease, or HIV-specific QoL measures—are tailored to capture symptoms and functional limitations unique to particular conditions (Glover et al., 2011; Namisango et al., 2007). Although these tools generally show stronger clinical sensitivity, many lack extensive validation in African populations. Several studies note inconsistencies in factor structures, challenges in linguistic adaptation, and reduced responsiveness due to cultural variations in symptom reporting (Ngwira et al., 2021; Soto et al., 2015; Crawford, 2012). The distinction between generic and disease-specific tools is, therefore, essential, as their adequacy in Africa varies and depends on rigorous local validation.

However, owing to the need for high-quality, specifically designed questionnaires based on patient-reported outcomes (PROs) in clinical practice, the instruments are usually translated into different languages (Ware, 2000; Herdman et al., 2011). Evidence shows that the reliability and validity of an instrument are influenced by socioeconomic factors, such as education, literacy, and rural or urban living, which were often associated with populations’ cultural backgrounds and historical racial inequalities (Wissing et al., 2010; O’Keefe and Wood, 1996; Mullin et al., 2000; Nelson et al., 2020).

The HRQoL can be measured using generic or disease-specific instruments. Generic tools such as the SF-12, SF-36, EQ-5D, and WHOQOL-BREF capture broad aspects of physical, psychological, and social wellbeing and allow comparison across different diseases and populations. However, evidence from African studies shows that although these tools often demonstrate acceptable reliability, several items may not fully align with local cultural norms, particularly in domains related to social relationships, spirituality, and environmental context (Olsen et al., 2013; Gladstone et al., 2008). Disease-specific instruments, such as the EORTC QLQ-C30 for cancer or diabetes-specific QoL scales, provide more clinically sensitive assessments. However, many have undergone limited validation in African settings, with challenges reported in linguistic adaptation, conceptual equivalence, and responsiveness (Naamala et al., 2021; Marsh and Truter, 2021). This distinction matters because the adequacy of each type of tool in Africa depends on rigorous and context-specific validation.

Validating HRQoL instruments typically involves assessing their reliability, validity, and responsiveness, as well as ensuring cultural and linguistic appropriateness. This is especially important in Africa, where cultural norms, language diversity, and shifting health burdens from infectious to chronic non-communicable diseases may influence how individuals interpret and respond to HRQoL items (Marsh and Truter, 2021). Despite increasing use of these tools, synthesized evidence on their validation in African populations is lacking, underscoring the need for a systematic review to map existing instruments, highlight gaps, and guide future adaptation or development.

To support this assessment, this review uses the COSMIN (Consensus-based Standards for the Selection of Health Measurement Instruments) framework, which provides internationally recognized criteria for evaluating the methodological quality of studies on PROMs (Mokkink et al., 2016). Although alternative guidelines exist, such as ISOQOL standards or the FDA PRO guidance, COSMIN offers the most comprehensive and structured approach for evaluating psychometric properties, making it particularly suitable for this review (Lorente et al., 2020). Despite the growing use of HRQoL instruments in African health research, there remains limited consolidated evidence on how these tools have been developed, adapted, and psychometrically validated for use across the continent’s diverse cultural and linguistic contexts. No prior systematic review has comprehensively synthesized this evidence, even though such information is essential for ensuring that PROMs are conceptually appropriate, reliable, and meaningful for African populations. Therefore, the objective of this systematic review is to identify and critically appraise all studies that have developed, adapted, or validated generic or disease-specific HRQoL instruments for African populations. Guided by the PROSPERO-registered protocol (CRD42025639055), the review aims to answer the following research question: “Which HRQoL measurement tools have been validated or culturally adapted for use in African populations, and what is the quality of the evidence supporting their psychometric properties and contextual relevance?” This study also seeks to highlight the methodological strengths and limitations of the included studies to inform future research and to promote more robust and culturally appropriate HRQoL measurement across African settings.

## Methods

### Design

This study was conducted as a systematic review to identify, evaluate, and document HRQoL measurement tools developed or validated for use in African populations. This systematic review assessed the psychometric properties, cultural adaptation, and validation of HRQoL tools across diverse populations in Africa. The review adhered to the Preferred Reporting Items for Systematic Reviews and Meta-Analyses (PRISMA) guidelines. The protocol was formally registered with the International Prospective Register of Systematic Reviews (PROSPERO), registration number CRD42025639055.

### Eligibility criteria

Studies were eligible for inclusion if they met the following criteria:

- Studies conducted among children or adult populations residing in Africa.
- Studies that focused on the development, cross-cultural or linguistic adaptation, or psychometric validation of an HRQoL tool. Adaptation was defined as any modification made to an existing HRQoL instrument to improve its cultural, linguistic, or contextual relevance to an African setting. This includes translation, back-translation, and pilot testing. Validation was defined as the evaluation of one or more psychometric properties of the HRQoL instrument, such as reliability (e.g., internal consistency and test–retest), construct validity, criterion validity, responsiveness, or factor structure, based on the COSMIN guidelines.
- Quantitative, qualitative, cross-sectional, longitudinal, and mixed-method studies.
- Peer-reviewed primary studies with sufficient methodological detail from 1 January 2015 to 1 January 2025.

### Exclusion criteria

The study’s exclusion criteria were as follows:

- Studies that exclusively focused on non-human populations.
- Studies that used HRQoL tools without developing, adapting, or validating them for African populations.
- Unpublished theses and retrospective analyses of secondary datasets.

### Search strategy

A comprehensive literature search was conducted across PubMed, Web of Science, Scopus, and gray literature sources. Additional sources included the reference lists of relevant articles. The search was designed to retrieve studies published to date, using a combination of MeSH terms and free-text keywords related to HRQoL measurement tools, their development, validation, and use in Africa. Boolean operators (AND/OR) and truncation (*) were applied where necessary to refine the search.

The primary search terms included: (*“tool” OR “instrument*” OR “scale*” OR “questionnaire*” OR “measure*” OR “assessment tool*” OR “survey*”) AND (“health-related quality of life” OR “HRQoL” OR “QoL” OR “quality of life” OR “health preference*”) AND (“measurement” OR “assessment” OR “evaluation” OR “validation” OR “development”) AND (“Africa” OR “Sub-Saharan Africa” OR “African countries” OR “African region”)**. The search was conducted without language restrictions, but studies had to meet specific eligibility criteria to be included. The full search strategy from gray literature and databases (PubMed, Web of Science, and Scopus) is provided in Supplementary File 1.

### Study selection and screening

The search results were imported into Rayyan, a web-based tool for systematic reviews, where duplicate records were automatically removed after being assessed by AI. Title and abstract screening were independently conducted by EJU and CNI, with conflicts resolved by the third reviewer, AI. Similarly, full-text screening was performed by EJU and CNI, with discrepancies resolved by AI.

**Figure 1:**
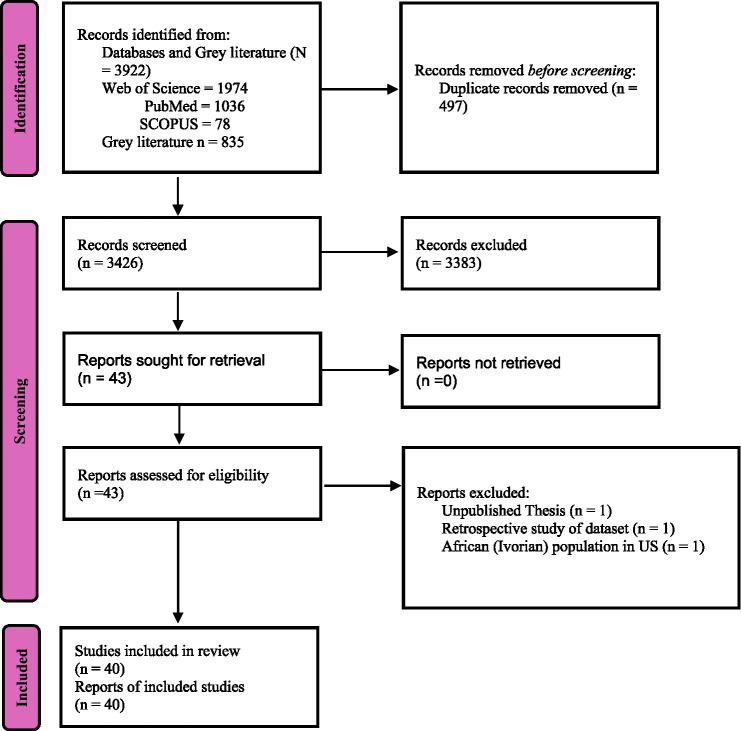
PRISMA flowchart for HRQoL tools validated for African population.

The study selection process is illustrated in Figure 1.

### Data extraction

A structured data extraction form was developed and managed using Microsoft Excel based on the study objectives and COSMIN guidelines. An artificial intelligence (AI) tool was also used to assist in data extraction. Any discrepancies in extracted data were discussed and resolved by consensus. The form was piloted on a random sample of three included studies to ensure clarity and completeness. Necessary revisions were made before full-scale data extraction. Two independent reviewers (EJU and CNI) extracted the following details:

- Study characteristics (authors, year, country, design).
- Participant characteristics (sample size, age group, setting).
- Tool characteristics (name, type, language, mode of administration, domains, validation process).
- Key findings, psychometric properties, and policy implications.

Any discrepancies in the extracted data were discussed and resolved by consensus, and when needed, a third reviewer (AI) acted as an arbitrator. This dual-coding and consensus-based approach ensured the reliability of the data extraction process. A quality assessment of the 40 included articles was conducted using the 8-item checklist for analytical cross-sectional studies by the Joanna Briggs Institute. Each of the eight questions was used to appraise the articles using the options “yes,” “no,” “unclear,” and “not applicable” (Supplementary File 2).

### Data synthesis

A narrative synthesis was conducted to summarize findings across studies. We conducted the synthesis using AI, categorizing the HRQoL tools into generic and disease-specific instruments and identifying trends in validation, adaptation, and psychometric evaluation. Quantitative metrics, including frequency distributions and proportions, were reported to enhance clarity.

Findings were structured according to the characteristics of the measurement tools, the characteristics of the first authors of the included studies, the psychometric properties of the tools, and their application across African settings.

## Results

A total of 3,922 records were retrieved from databases, of which 3,425 remained after duplicate removal. After title and abstract screening, 43 articles were retained for full-text review. Three studies were excluded at this stage: one unpublished thesis, one retrospective dataset analysis, and one study on an Ivorian population residing in the United States. Ultimately, 40 studies were included in this systematic review, with the majority (82.5%, *n* = 33) employing a cross-sectional design alone (Colbourn et al., 2012; Bowden et al., 2002; Namisango et al., 2007; Van Biljon et al., 2015; Reba et al., 2019; Younsi and Chakroun, 2014; Ibrahim et al., 2020; Jikamo et al., 2021; Mbada et al., 2015; Mgbeojedo et al., 2022; Muhye and Fentahun, 2023; Ravens-Sieberer et al., 2010; Ehab et al., 2021; Duracinsky et al., 2012; Gqada et al., 2021; Onagbiye et al., 2018; Uwizihiwe et al., 2022; Kidayi et al., 2023; Brandt et al., 2016; Borissov et al., 2022; Kondo et al., 2023; El Fakir et al., 2014a; Olasehinde et al., 2024; Nkurunziza et al., 2016; Farid et al., 2023; El Fakir et al., 2014b; Odetunde et al., 2020; Araya et al., 2019; El Alami et al., 2021; Osman et al., 2018; Smith and Morris-Eyton, 2023; Westmoreland et al., 2018; Guermazi et al., 2012), while only 15% (*n* = 6) used a longitudinal approach alone (Scott et al., 2017; Ohrnberger et al., 2020; Okello et al., 2018; Owolabi, 2010; Kulich et al., 2008; Getu et al., 2022; Gadisa et al., 2019). Notably, 80.0% (*n* = 32) of the studies focused on the translation and cultural adaptation of tools to align with local contexts (Colbourn et al., 2012; Bowden et al., 2002; Van Biljon et al., 2015; Reba et al., 2019; Ibrahim et al., 2020; Jikamo et al., 2021; Mbada et al., 2015; Mgbeojedo et al., 2022; Muhye and Fentahun, 2023; Ehab et al., 2021; Duracinsky et al., 2012; Gqada et al., 2021; Onagbiye et al., 2018; Uwizihiwe et al., 2022; Kidayi et al., 2023; Brandt et al., 2016; Borissov et al., 2022; El Fakir et al., 2014a; Olasehinde et al., 2024; Nkurunziza et al., 2016; Farid et al., 2023; El Fakir et al., 2014b; Odetunde et al., 2020; Araya et al., 2019; El Alami et al., 2021; Osman et al., 2018; Smith and Morris-Eyton, 2023; Westmoreland et al., 2018; Guermazi et al., 2012; Kulich et al., 2008; Gadisa et al., 2019).

Geographically, South Africa (Van Biljon et al., 2015; Gqada et al., 2021; Brandt et al., 2016; Scott et al., 2017) and Ethiopia (Reba et al., 2019; Jikamo et al., 2021; Muhye and Fentahun, 2023; Araya et al., 2019; Getu et al., 2022; Gadisa et al., 2019) were the most represented countries, contributing 10.0% (*n* = 4) and 15% (*n* = 6) of the studies, respectively. Among studies on disease-specific tools, breast cancer and diabetes (Reba et al., 2019; Ehab et al., 2021; Uwizihiwe et al., 2022; Kidayi et al., 2023; El Fakir et al., 2014a; Olasehinde et al., 2024; Getu et al., 2022; Gadisa et al., 2019) were the most frequently studied conditions, representing 20% (*n* = 8) of the included studies (Table 1).

**Table 1:** Descriptive characteristics of the included studies in the review.

Ref. No.	Author(s)	Year	Title	Aim of the study	Study design
1	Van Biljon et al. (2015)	2015	A partial validation of the WHOQOL-OLD in a sample of older people in South Africa	Partial validation (i.e., the assessment of the factor structure and the internal consistency reliability) of the WHOQOL-OLD and its shorter versions	Cross-sectional (quantitative)
2	Smith and Morris-Eyton (2023)	2023	Development, validation and reliability of the Smith Toolkit for Integrated Health Related Quality of Life (STI-HRQoL)	Develop a toolkit that could assess the HRQoL in patients with hypertension, type 2 diabetes, and cardiovascular disease	Cross-sectional (mixed methods)
3	Reba et al. (2019)	2019	Validity and reliability of the Amharic version of the World Health Organization’s Quality of Life Questionnaire (WHOQOLBREF) in patients with diagnosed type 2 diabetes in Felege Hiwot Referral Hospital, Ethiopia	Validate the Amharic version of WHOQOL-BREF, which is designed for measuring QoL of people with diagnosed type 2 diabetes in Felege Hiwot Referral Hospital	Cross-sectional (quantitative)
4	Westmoreland et al. (2018)	2018	Translation, psychometric validation, and baseline results of the Patient-Reported Outcomes Measurement Information System (PROMIS) pediatric measures to assess health-related quality of life of patients with pediatric lymphoma in Malawi	Translate and culturally validate PROMIS pediatric measures into Chichewa and report on HRQoL at the time of diagnosis among pediatric patients with lymphoma in Malawi	Cross-sectional (mixed methods)
5	Younsi and Chakroun (2014)	2014	Measuring health-related quality of life: psychometric evaluation of the Tunisian version of the SF-12 health survey	Examine the psychometric properties of the Tunisian version of SF-12 in terms of the measurement and conceptual model, sensitivity, “known groups” construct validity, convergent validity, and hence to increase confidence in using the SF-12 in Tunisian studies as an alternative to the more time-demanding SF-36	Cross-sectional (quantitative)
6	Ibrahim et al. (2020)	2020	The Hausa 12-item short-form health survey (SF-12): translation, cross-cultural adaptation and validation in mixed urban and rural Nigerian populations with chronic low back pain	Translate and cross-culturally adapt the SF-12 into the Hausa language and test its psychometric properties in mixed urban and rural Nigerian populations with chronic LBP	Cross-sectional (quantitative)
7	Jikamo et al. (2021)	2021	Cultural adaptation and validation of the Sidamic version of the World Health Organization Quality-of-Life-BREF Scale measuring the quality of life of women with severe preeclampsia in southern Ethiopia, 2020	Translate, culturally adapt, and test the reliability and validity of the WHOQOL-BREF when measuring the quality of life of women with severe preeclampsia in southern Ethiopia	Cross-sectional (quantitative)
8	Mbada et al. (2015)	2015	Translation, cross-cultural adaptation and psychometric evaluation of Yoruba version of the short-form 36 health survey	Cross-culturally adapt the SF-36 into the Yoruba language and determine its reliability and validity	Cross-sectional (quantitative)
9	Namisango et al. (2007)	2007	Validation of the Missoula-Vitas Quality of-Life Index Among Patients with Advanced AIDS in Urban Kampala, Uganda	Explore the validity and reliability of the MVQOLI in terminally ill AIDS patients receiving palliative care in Uganda, Africa	Cross-sectional (quantitative)
10	Colbourn et al. (2012)	2012	Development, reliability and validity of the Chichewa WHOQOL-BREF in adults in Lilongwe, Malawi	Describes the translation from English to Chichewa, adaptation, and piloting process that constitutes the validation of the WHOQOL-BREF in Malawi.	Cross-sectional (quantitative)
11	Guermazi et al. (2012)	2012	Translation in Arabic, adaptation and validation of the SF-36 Health Survey for use in Tunisia	Translate into Tunisian Arabic and validate the SF-36 in a Tunisian population	Cross-sectional (mixed methods)
12	Mgbeojedo et al. (2022)	2022	IGBO version of the Older People’s Quality of Life Questionnaire (OPQOL-35) is valid and reliable: cross-cultural adaptation and validation	Translate, cross-culturally adapt, and psychometrically evaluate the OPQOL-35 among the Igbo older adult population in Enugu State	Cross-sectional (quantitative)
13	Muhye and Fentahun (2023)	2023	Validation of quality-of-life assessment tool for Ethiopian old age people	Translate and validate the WHOQOL-OLD tool for Ethiopian older adults	Cross-sectional (quantitative)
14	Scott et al. (2017)	2017	The use of the EQ-5D-Y health related quality of life outcome measure in children in the Western Cape, South Africa: psychometric properties, feasibility and usefulness – a longitudinal, analytical study	Investigate the psychometric properties of the EQ-5D-Y when used to assess the HRQoL of children with different health states	Longitudinal (cohort study)
15	Ravens-Sieberer et al. (2010)	2010	Feasibility, reliability, and validity of the EQ-5D-Y: results from a multinational study	Examine the feasibility, reliability, and validity of the newly developed EQ-5D-Y	Cross-sectional (quantitative), with additional test–retest procedures
16	Ehab et al. (2021)	2021	Cultural adaptation and validation of the EORTC QLQ-BR45 to assess health-related quality of life of breast cancer patients	Perform cultural adaptation, pilot testing, and assessment of the psychometric properties of the Egyptian Arabic translation of the EORTC QLQBR45 module on Egyptian breast cancer patients	Cross-sectional (quantitative)
17	Duracinsky et al. (2012)	2012	Psychometric validation of the PROQOL-HIV questionnaire, a new health-related quality of life instrument–specific to HIV disease	Showed the psychometric validation of the PROQOL-HIV instrument using data simultaneously collected in eight countries	Cross-sectional (quantitative)
18	Gqada et al. (2021)	2021	Translation and linguistic validation of the EORTC QLQ-PAN26 questionnaire for assessment of health-related quality of life in patients with pancreatic cancer and chronic pancreatitis into isiXhosa and Afrikaans	Translated and validated the EORTC QLQPAN26 questionnaire into isiXhosa and Afrikaans	Cross-sectional (quantitative)
19	Onagbiye et al. (2018)	2018	Validity and reliability of the Setswana translation of the Short Form-8 health-related quality of life health survey in adults	Explored the feasibility and reliability of the Setswana translation of the HRQoL Short Form-8 (SF-8) among Setswana-speaking adults	Cross-sectional (quantitative)
20	Ohrnberger et al. (2020)	2020	Validation of the SF12 mental and physical health measure for the population from a low-income country in sub-Saharan Africa	Computed and validated the SF-12 for the Malawian population	Longitudinal study (quantitative)
21	Okello et al. (2018)	2018	Validation of heart failure quality of life tool and usage to predict all-cause mortality in acute heart failure in Uganda: The Mbarara heart failure registry (MAHFER)	Validated the Kansas City Cardiomyopathy Questionnaire (KCCQ) and evaluated its use as a predictor of 3-month all-cause mortality among heart failure participants in rural Uganda	Longitudinal study (quantitative)
22	Uwizihiwe et al. (2022)	2022	Cultural adaptation and psychometric evaluation of the Kinyarwanda version of the diabetes-39 (D-39) questionnaire	Translation and cultural adaptation of the Diabetes-39 (D-39) questionnaire into Kinyarwanda and its psychometric properties among diabetic patients in Rwanda	Cross-sectional (quantitative)
23	Owolabi (2010)	2010	Psychometric properties of the HRQOLISP-40: a novel, shortened multiculturally valid holistic stroke measure	Determined the psychometric properties of a shortened version of the HRQOLISP in multicultural transnational populations	Longitudinal study (quantitative)
24	Kidayi et al. (2023)	2023	Cross-cultural adaptation and psychometric properties of the Swahili version of the European Organization for Research and Treatment of Cancer (EORTC) QLQ-BR45 among breast cancer patients in Tanzania	Determined the validity, reliability, and psychometric properties of the Swahili version of EORTC QLQ-BR45 among women with breast cancer in Tanzania	Cross-sectional (quantitative)
25	Brandt et al. (2016)	2016	Validation of the prolapse quality-of-life questionnaire (P-QoL): an Afrikaans version in a South African population	Validated an Afrikaans version of the P-QoL in a South African population	Cross-sectional (quantitative)
26	Kulich et al. (2008)	2008	Reliability and validity of the Gastrointestinal Symptom Rating Scale (GSRS) and Quality of Life in Reflux and Dyspepsia (QOLRAD) questionnaire in dyspepsia: a six-country study	Documented the psychometric characteristics of the Gastrointestinal Symptom Rating Scale (GSRS) and the Quality of Life in Reflux and Dyspepsia questionnaire (QOLRAD) in Afrikaans, German, Hungarian, Italian, Polish, and Spanish patients with dyspepsia	Longitudinal study (quantitative)
27	Borissov et al. (2022)	2021	Adaptation and validation of two autism-related measures of skills and quality of life in Ethiopia	Culturally adapt and validate two questionnaires for use in Ethiopia: The Autism Treatment Evaluation Checklist and the Pediatric Quality of Life Inventory™ Family Impact Module	Cross-sectional (quantitative)
28	Kondo et al. (2023)	2023	Validation of Kiswahili version of WHOQOL-HIV BREF questionnaire among people living with HIV/AIDS in Tanzania – a cross-sectional study	Assess the validity and reliability of the Kiswahili version of WHOQOL-HIV BREF among PLWHA in Tanzania	Cross-sectional (quantitative)
29	El Fakir et al. (2014a)	2014	The European organization for research and treatment of cancer quality of life questionnaire-BR 23 breast cancer-specific quality of life questionnaire: psychometric properties in a Moroccan sample of breast cancer patients	Translate and adapt the original version of the European Organization for Research and Treatment of Cancer (EORTC) Breast Cancer-Specific Quality of Life Questionnaire (EORTC QLQ-BR23) from English to Moroccan Arabic language, to refine its terminology and to adapt it to the Moroccan culture	Cross-sectional (quantitative)
30	Getu et al. (2022)	2022	Translation and validation of the EORTC QLQ-BR45 among Ethiopian breast cancer patients	Translate, validate, and assess the psychometric properties of the EORTC QLQBR45 among breast cancer patients in Ethiopia	Longitudinal study (quantitative)
31	Olasehinde et al. (2024)	2024	Translation and psychometric assessment of the mastectomy module of the BREAST-Q questionnaire for use in Nigeria	Translate and assess the psychometric properties of the mastectomy module of the BREAST-Q for use in Nigeria	Cross-sectional (quantitative)
32	Nkurunziza et al. (2016)	2016	Validation of the Kinyarwanda-version Short-Form Leeds Dyspepsia Questionnaire and Short-Form Nepean Dyspepsia Index to assess dyspepsia prevalence and quality-of-life impact in Rwanda	Develop and validate Kinyarwanda versions of the Short-Form Leeds Dyspepsia Questionnaire (SF-LDQ) and the Short-Form Nepean Dyspepsia Index (SF-NDI) to measure the frequency and severity of dyspepsia and associated quality-of-life impact in Rwanda	Cross-sectional (quantitative)
33	Farid et al. (2023)	2023	Cross-cultural adaptation and validation of a self-reporting tool to assess health-related quality of life for Egyptians with extremity bone sarcomas in childhood or adolescence	Cross-cultural adaptation and validation of the pediatric Toronto Extremity Salvage Score (pTESS) and Toronto Extremity Salvage Score (TESS) to assess the functional outcome for Egyptian children and adult survivors following surgeries of extremity bone sarcomas	Cross-sectional (quantitative)
34	El Fakir et al. (2014b)	2012	Validation of the Skindex-16 questionnaire in patients with skin diseases in Morocco	To translate and adapt the original version of the Skindex-16 questionnaire from English to Moroccan Arabic language, refining its terms and adapting it to Moroccan culture	Cross-sectional (quantitative)
35	Odetunde et al. (2020)	2020	Cross-cultural adaptation and validation of the Igbo language version of the stroke-specific quality of life scale 2.0	Cross-culturally adapting and assessing the validity and reliability of the Igbo version of the SS-QoL	Cross-sectional (quantitative)
36	Araya et al. (2019)	2019	Reliability and validity of the Amharic version of European Organization for Research and Treatment of cervical cancer module for the assessment of health-related quality of life in women with cervical cancer in Addis Ababa, Ethiopia	Assess the psychometric properties of the tool among Ethiopian cervical cancer patients	Cross-sectional (quantitative)
37	El Alami et al. (2021)	2021	Psychometric validation of the Moroccan version of the EORTC QLQ-C30 in colorectal Cancer patients: cross-sectional study and systematic literature review	Assess the validity and reliability of the Moroccan Arabic Dialectal version of the European Organization for Research and Treatment of Cancer (EORTC) Quality of Life Core Questionnaire (QLQ-C30) in patients with colorectal cancer	Cross-sectional (quantitative)
38	Gadisa et al. (2019)	2019	Reliability and validity of Amharic version of EORTC QLQ-C30 and QLQ-BR23 modules for assessing health-related quality of life among breast cancer patients in Ethiopia	Assess the psychometric properties of the tools among Ethiopian breast cancer patients	Longitudinal Study (quantitative)
39	Osman et al. (2018)	2018	Validation and comparison of the Arabic versions of GOHAI and OHIP-14 in patients with and without denture experience	Compare and assess the validation of two quality of life measures, the Oral Health Impact Profile-14 (OHIP-14) and Geriatric Oral Health Assessment Index (GOHAI), in patients with and without previous denture experience	Cross-sectional (quantitative)
40	Bowden et al. (2002)	2002	Methods for pre-testing and piloting survey questions: illustrations from the KENQOL survey of health-related quality of life	develop a culturally relevant generic measure of ‘health’ to measure the impact of interventions designed to reduce disease and/or improve health in Kenya	Cross-sectional (qualitative)

WHOQOL-OLD, World Health Organization quality of life for older adults; STI-HRQoL, Smith Toolkit for Integrated Health-Related Quality of Life; WHOQOL-BREF, World Health Organization Quality of Life (brief version); HRQoL, health-related quality of life; PROMIS, Patient-Reported Outcomes Measurement Information System; SF-12, 12-item short-form health survey; SF-36, 36-item short-form health survey; MVQOLI, Missoula-Vitas Quality of-Life Index; AIDS, acquired immunodeficiency syndrome; OPQOL-35, Older People’s Quality of Life Questionnaire (35 items); LBP, lower back pain; QoL, quality of life. EQ-5D-Y, EuroQol Group 5-dimension for children and adolescents; EORTC QLQ-BR45, European Organization for Research and Treatment of Cancer—quality of life questionnaire for breast cancer patients (45 items); PROQOL-HIV, Patient-Reported Outcomes Quality of Life–HIV questionnaire; EORTC QLQ-PAN26, European Organization for Research and Treatment of Cancer—quality of life questionnaire for patients with pancreatic cancer and chronic pancreatitis (26 items); D-39, Diabetes (39 items); HRQOLISP-40, health-related quality of life in stroke patients (40 items); P-QoL, prolapse quality-of-life questionnaire; GSRS, Gastrointestinal Symptom Rating Scale; QOLRAD, Quality of Life in Reflux and Dyspepsia; KCCQ, Kansas City Cardiomyopathy Questionnaire; SF-8, 8-item short-form survey. WHOQOL-HIV BREF, World Health Organization quality of life for HIV patients; EORTC QLQ-C30, European Organization for Research and Treatment of Cancer—quality of life questionnaire for Colorectal cancer patients (30 items); GOHAI, Geriatric Oral Health Assessment Index; OHIP-14, Oral Health Impact Profile-14; QLQ-C30, Quality of Life Core Questionnaire in patients with colorectal cancer (30 items); SS-QoL, stroke-specific quality of life scale; pTESS, Toronto Extremity Salvage Score; TESS, Toronto Extremity Salvage Score; SF-LDQ, Short-Form Leeds Dyspepsia Questionnaire; SF-NDI, Short-Form Nepean Dyspepsia Index; BREAST-Q, Breast Questionnaire; KENQOL, Kenyan quality of life.

### Quality assessment of the included articles using the Joanna Briggs Institute checklist

The vast majority of studies demonstrated robust practices, with 34 (85%) clearly defining their inclusion criteria (Colbourn et al., 2012; Namisango et al., 2007; Van Biljon et al., 2015; Reba et al., 2019; Younsi and Chakroun, 2014; Ibrahim et al., 2020; Jikamo et al., 2021; Mbada et al., 2015; Mgbeojedo et al., 2022; Muhye and Fentahun, 2023; Ravens-Sieberer et al., 2010; Ehab et al., 2021; Onagbiye et al., 2018; Uwizihiwe et al., 2022; Kidayi et al., 2023; Brandt et al., 2016; Borissov et al., 2022; Kondo et al., 2023; Olasehinde et al., 2024; Nkurunziza et al., 2016; Farid et al., 2023; El Fakir et al., 2014b; Odetunde et al., 2020; Araya et al., 2019; El Alami et al., 2021; Osman et al., 2018; Smith and Morris-Eyton, 2023; Scott et al., 2017; Okello et al., 2018; Owolabi, 2010; Kulich et al., 2008; Getu et al., 2022; Gadisa et al., 2019) and 33 (82.5%) providing a detailed description of the study subjects and setting (Colbourn et al., 2012; Namisango et al., 2007; Van Biljon et al., 2015; Reba et al., 2019; Younsi and Chakroun, 2014; Ibrahim et al., 2020; Jikamo et al., 2021; Mbada et al., 2015; Mgbeojedo et al., 2022; Muhye and Fentahun, 2023; Ravens-Sieberer et al., 2010; Ehab et al., 2021; Onagbiye et al., 2018; Uwizihiwe et al., 2022; Kidayi et al., 2023; Brandt et al., 2016; Borissov et al., 2022; Kondo et al., 2023; Olasehinde et al., 2024; Nkurunziza et al., 2016; Farid et al., 2023; El Fakir et al., 2014b; Odetunde et al., 2020; Araya et al., 2019; El Alami et al., 2021; Smith and Morris-Eyton, 2023; Scott et al., 2017; Okello et al., 2018; Owolabi, 2010; Kulich et al., 2008; Getu et al., 2022; Gadisa et al., 2019). Measurement and analysis were particularly strong, as nearly all studies, 37 (97.5%), reported measuring outcomes in a valid and reliable way and employing appropriate statistical analysis (Colbourn et al., 2012; Bowden et al., 2002; Namisango et al., 2007; Van Biljon et al., 2015; Reba et al., 2019; Younsi and Chakroun, 2014; Ibrahim et al., 2020; Jikamo et al., 2021; Mbada et al., 2015; Mgbeojedo et al., 2022; Muhye and Fentahun, 2023; Ravens-Sieberer et al., 2010; Ehab et al., 2021; Duracinsky et al., 2012; Gqada et al., 2021; Onagbiye et al., 2018; Uwizihiwe et al., 2022; Kidayi et al., 2023; Brandt et al., 2016; Borissov et al., 2022; Kondo et al., 2023; Olasehinde et al., 2024; Nkurunziza et al., 2016; Farid et al., 2023; El Fakir et al., 2014b; Odetunde et al., 2020; Araya et al., 2019; El Alami et al., 2021; Osman et al., 2018; Smith and Morris-Eyton, 2023; Scott et al., 2017; Ohrnberger et al., 2020; Okello et al., 2018; Owolabi, 2010; Kulich et al., 2008; Getu et al., 2022; Gadisa et al., 2019). However, the handling of confounding factors was a notable exception. Of the 33 studies for which this criterion was applicable, only 8 (24.2%) adequately identified potential confounders (Van Biljon et al., 2015; Reba et al., 2019; Ibrahim et al., 2020; Mbada et al., 2015; Borissov et al., 2022; Osman et al., 2018; Smith and Morris-Eyton, 2023; Getu et al., 2022) (see Supplementary Tables 2a–c).

### Characteristics of first authors of the included studies

Among the 40 included studies, 22 (55%) of the first authors were men (Colbourn et al., 2012; Bowden et al., 2002; Younsi and Chakroun, 2014; Ibrahim et al., 2020; Jikamo et al., 2021; Mbada et al., 2015; Muhye and Fentahun, 2023; Duracinsky et al., 2012; Uwizihiwe et al., 2022; Kidayi et al., 2023; Borissov et al., 2022; Kondo et al., 2023; Olasehinde et al., 2024; Nkurunziza et al., 2016; El Alami et al., 2021; Guermazi et al., 2012; Ohrnberger et al., 2020; Okello et al., 2018; Owolabi, 2010; Kulich et al., 2008; Getu et al., 2022; Gadisa et al., 2019), while 13 (32.5%) were women (Namisango et al., 2007; Mgbeojedo et al., 2022; Ravens-Sieberer et al., 2010; Ehab et al., 2021; Brandt et al., 2016; El Fakir et al., 2014a; Farid et al., 2023; El Fakir et al., 2014b; Odetunde et al., 2020; Osman et al., 2018; Smith and Morris-Eyton, 2023; Westmoreland et al., 2018; Scott et al., 2017), indicating a gender disparity in authorship of HRQoL research in Africa. The majority of the first authors (*n* = 32, 80.0%) were affiliated with African institutions (Colbourn et al., 2012; Namisango et al., 2007; Van Biljon et al., 2015; Reba et al., 2019; Younsi and Chakroun, 2014; Ibrahim et al., 2020; Jikamo et al., 2021; Mbada et al., 2015; Mgbeojedo et al., 2022; Muhye and Fentahun, 2023; Ehab et al., 2021; Gqada et al., 2021; Onagbiye et al., 2018; Kidayi et al., 2023; Brandt et al., 2016; Kondo et al., 2023; El Fakir et al., 2014a; Olasehinde et al., 2024; Nkurunziza et al., 2016; Farid et al., 2023; El Fakir et al., 2014b; Odetunde et al., 2020; Araya et al., 2019; El Alami et al., 2021; Osman et al., 2018; Smith and Morris-Eyton, 2023; Westmoreland et al., 2018; Guermazi et al., 2012; Scott et al., 2017; Okello et al., 2018; Gadisa et al., 2019), while 7 (17.5%) were affiliated with institutions outside Africa (Ravens-Sieberer et al., 2010; Duracinsky et al., 2012; Uwizihiwe et al., 2022; Borissov et al., 2022; Ohrnberger et al., 2020; Kulich et al., 2008; Getu et al., 2022; Bowden, 2002). The United Kingdom (Borissov et al., 2022; Ohrnberger et al., 2020; Bowden, 2002) (*n* = 3, 7.5%) and France (Duracinsky et al., 2012) (*n* = 1, 2.5%) were represented among non-African affiliations. South Africa (Van Biljon et al., 2015; Gqada et al., 2021; Onagbiye et al., 2018; Brandt et al., 2016; Smith and Morris-Eyton, 2023; Scott et al., 2017) (*n* = 6, 15.0%), Nigeria (Ibrahim et al., 2020; Mbada et al., 2015; Mgbeojedo et al., 2022; Olasehinde et al., 2024; Odetunde et al., 2020; Owolabi, 2010) (*n* = 6, 15.0%), and Ethiopia (Reba et al., 2019; Jikamo et al., 2021; Muhye and Fentahun, 2023; Araya et al., 2019; Gadisa et al., 2019) (*n* = 5, 12.5%) had the highest representation of first-author institutional affiliations. Dual institutional affiliations were observed in 14 (35.0%) of the studies, reflecting interdisciplinary and cross-institutional research collaborations (Colbourn et al., 2012; Ibrahim et al., 2020; Jikamo et al., 2021; Mbada et al., 2015; Mgbeojedo et al., 2022; Duracinsky et al., 2012; Gqada et al., 2021; Uwizihiwe et al., 2022; Olasehinde et al., 2024; El Alami et al., 2021; Westmoreland et al., 2018; Guermazi et al., 2012; Okello et al., 2018; Getu et al., 2022) (see Supplementary Tables 3a–c).

### Descriptive characteristics of sample sizes and populations whose HRQoL were assessed

Most studies (77.5%, *n* = 31) focused on adult populations aged 18 years and above (Colbourn et al., 2012; Namisango et al., 2007; Reba et al., 2019; Younsi and Chakroun, 2014; Ibrahim et al., 2020; Jikamo et al., 2021; Mbada et al., 2015; Ehab et al., 2021; Duracinsky et al., 2012; Gqada et al., 2021; Uwizihiwe et al., 2022; Kidayi et al., 2023; Brandt et al., 2016; Kondo et al., 2023; El Fakir et al., 2014a; Olasehinde et al., 2024; Nkurunziza et al., 2016; Farid et al., 2023; El Fakir et al., 2014b; Odetunde et al., 2020; Araya et al., 2019; El Alami et al., 2021; Osman et al., 2018; Smith and Morris-Eyton, 2023; Guermazi et al., 2012; Ohrnberger et al., 2020; Okello et al., 2018; Owolabi, 2010; Kulich et al., 2008; Getu et al., 2022; Gadisa et al., 2019). In comparison, only 7.5% (*n* = 3) included children and adolescents alongside the adult population (Mgbeojedo et al., 2022; Ravens-Sieberer et al., 2010; Westmoreland et al., 2018), suggesting a significant emphasis on adult HRQoL in African settings.

Sample sizes varied considerably, with 80.0% (*n* = 32) of studies enrolling fewer than 300 participants (Namisango et al., 2007; Van Biljon et al., 2015; Ibrahim et al., 2020; Jikamo et al., 2021; Mgbeojedo et al., 2022; Muhye and Fentahun, 2023; Ehab et al., 2021; Onagbiye et al., 2018; Uwizihiwe et al., 2022; Kidayi et al., 2023; Brandt et al., 2016; Borissov et al., 2022; Kondo et al., 2023; El Fakir et al., 2014a; Olasehinde et al., 2024; Nkurunziza et al., 2016; Farid et al., 2023; El Fakir et al., 2014b; Odetunde et al., 2020; Araya et al., 2019; El Alami et al., 2021; Osman et al., 2018; Smith and Morris-Eyton, 2023; Westmoreland et al., 2018; Guermazi et al., 2012; Scott et al., 2017; Okello et al., 2018; Owolabi, 2010; Kulich et al., 2008; Getu et al., 2022; Gadisa et al., 2019; Gqada et al., 2021), and only 7.5% (*n* = 3) involving cohorts exceeding 1,000 participants (Younsi and Chakroun, 2014; Mbada et al., 2015; Ohrnberger et al., 2020).

Recruitment settings were predominantly hospital-based (Colbourn et al., 2012; Namisango et al., 2007; Van Biljon et al., 2015; Reba et al., 2019; Ibrahim et al., 2020; Jikamo et al., 2021; Mbada et al., 2015; Ehab et al., 2021; Duracinsky et al., 2012; Uwizihiwe et al., 2022; Kidayi et al., 2023; Brandt et al., 2016; Borissov et al., 2022; Kondo et al., 2023; El Fakir et al., 2014a; Olasehinde et al., 2024; Nkurunziza et al., 2016; Farid et al., 2023; El Fakir et al., 2014b; Odetunde et al., 2020; Araya et al., 2019; El Alami et al., 2021; Osman et al., 2018; Smith and Morris-Eyton, 2023; Westmoreland et al., 2018; Okello et al., 2018; Owolabi, 2010; Kulich et al., 2008; Getu et al., 2022; Gadisa et al., 2019; Gqada et al., 2021) (77.5%, *n* = 31), with only 22.5% (*n* = 9) conducted in community settings (Bowden et al., 2002; Younsi and Chakroun, 2014; Mgbeojedo et al., 2022; Muhye and Fentahun, 2023; Ravens-Sieberer et al., 2010; Onagbiye et al., 2018; Guermazi et al., 2012; Scott et al., 2017; Ohrnberger et al., 2020), indicating a potential bias toward healthcare facility-based populations.

Regional distribution revealed East Africa as the leading contributor (Colbourn et al., 2012; Bowden et al., 2002; Namisango et al., 2007; Reba et al., 2019; Jikamo et al., 2021; Muhye and Fentahun, 2023; Uwizihiwe et al., 2022; Kidayi et al., 2023; Borissov et al., 2022; Kondo et al., 2023; Nkurunziza et al., 2016; Araya et al., 2019; Westmoreland et al., 2018; Ohrnberger et al., 2020; Okello et al., 2018; Getu et al., 2022; Gadisa et al., 2019) (42.5%, *n* = 17), with West Africa (Ibrahim et al., 2020; Mbada et al., 2015; Mgbeojedo et al., 2022; Duracinsky et al., 2012; Olasehinde et al., 2024; Odetunde et al., 2020; Owolabi, 2010) (17.5%, *n* = 7) contributing the least proportion of studies. Gender-specific studies were limited, with only 17.5% (*n* = 7) exclusively targeting women, highlighting a notable gap in gender-focused HRQoL research (Jikamo et al., 2021; Kidayi et al., 2023; Brandt et al., 2016; El Fakir et al., 2014a; Olasehinde et al., 2024; Araya et al., 2019; Osman et al., 2018) (see Table 2).

**Table 2:** Descriptive characteristics of the participants in the included studies.

Ref. no.	Age group	Sample size	Facility	Country	Region
1.	Older adults	176	Hospital	South Africa	South
2.	Adults	257	Hospital	South Africa	South
3.	18 years and above	344	Hospital	Ethiopia	East
4.	Children between 5 and 18 years	54	Hospital	Malawi	East
5.	18 years and above	3,582	Community	Tunisia	North
6.	18–70 years	200	Hospital	Nigeria	West
7.	Adult women	264	Hospital	Ethiopia	East
8.	18–70 years	1,087	Hospital	Nigeria	West
9.	18–64 years	200	Hospital	Uganda	East
10.	Adults	309	Hospital	Malawi	East
11.	16–80 years	130	Community	Tunisia	North
12.	Older adults >65 years	115	Community	Nigeria	West
13.	60 years and above	180	Community	Ethiopia	East
14.	Adolescents (13–18 years)	224	Community	South Africa	South
15.	Children and adolescents	258	Community	South Africa (+5 non-Africans)	South
16.	18 and 65 years	74	Hospital	Egypt	North
17.	Adults	791	Hospital	Senegal (+7 non-Africans)	West
18.	Adults	13	Hospital	South Africa	South
19.	35–65 years	60	Community	South Africa	South
20.	15 years and older	2,838	community	Malawi	East
21.	13 years or greater	195	Hospital	Uganda	East
22.	Patients aged 21–80 years	309	Hospital	Uganda	East
23.	Adults	200 (Nigeria)	Hospital	Nigeria (+1 non-African)	West
24.	Adult women aged 18–70 years	422	Hospital	Tanzania	East
25.	Women 18–90 years	39	Hospital	South Africa	South
26.	Adults	108 (SA)	Hospital	South Africa (+5 non-African)	South
27.	Children between 2 and 9 years of age	300	Hospital	Ethiopia	East
28.	Aged 18 and above	73	Hospital	Tanzania	East
29.	At least 18 years	105	Hospital	Morocco	North
30.	18 years and above	248	Hospital	Ethiopia	East
31.	Women of different age categories	21	Hospital	Nigeria	West
32.	>17 years	200	Hospital	Rwanda	East
33.	Adult and children 8 years and above	233	Hospital	Egypt	North
34.	Above 18 years	120	Hospital	Morocco	North
35.	18 years and above	50	Hospital	Nigeria	West
36.	18 years and above	171	Hospital	Ethiopia	East
37.	18 years and above	120	Hospital	Morocco	North
38.	Above 18 years	146	Hospital	Ethiopia	East
39.	40 years and above	69	Hospital	Sudan	North
40.	Adults	550	Community	Kenya	East

### Characteristics of the HRQoL tools

A total of 34 tools were reported in the included studies. Of the 12 generic HRQoL instruments used in the studies, SF-12 (Younsi and Chakroun, 2014; Ibrahim et al., 2020; Ohrnberger et al., 2020) and WHOQOL-BREF (Colbourn et al., 2012; Reba et al., 2019; Jikamo et al., 2021) were reported in three studies each. Among the 22 disease-specific instruments, the different versions of the QoL in cancer tool, the EORTC QLQ, were validated in seven studies (Ehab et al., 2021; Kidayi et al., 2023; Araya et al., 2019; El Alami et al., 2021; Getu et al., 2022; Gadisa et al., 2019; Gqada et al., 2021), with the EORTC QLQ-C30 accounting for four of the studies (Kidayi et al., 2023; Araya et al., 2019; El Alami et al., 2021; Gadisa et al., 2019), emphasizing their widespread use in African health research. Most studies (82.5%, *n* = 33) translated tools into local languages, including Afrikaans, Chi Chew, Igbo, Yoruba, and Arabic, ensuring linguistic and cultural appropriateness (Colbourn et al., 2012; Bowden et al., 2002; Namisango et al., 2007; Van Biljon et al., 2015; Reba et al., 2019; Younsi and Chakroun, 2014; Ibrahim et al., 2020; Jikamo et al., 2021; Mbada et al., 2015; Mgbeojedo et al., 2022; Ehab et al., 2021; Duracinsky et al., 2012; Gqada et al., 2021; Onagbiye et al., 2018; Uwizihiwe et al., 2022; Kidayi et al., 2023; Brandt et al., 2016; Borissov et al., 2022; Kondo et al., 2023; El Fakir et al., 2014a; Olasehinde et al., 2024; Nkurunziza et al., 2016; Farid et al., 2023; Odetunde et al., 2020; Araya et al., 2019; El Alami et al., 2021; Osman et al., 2018; Westmoreland et al., 2018; Guermazi et al., 2012; Ohrnberger et al., 2020; Kulich et al., 2008; Getu et al., 2022; Gadisa et al., 2019). Administration methods varied, with 45% (*n* = 18) of the included studies adopting self-administration of 13 tools (Mgbeojedo et al., 2022; Gqada et al., 2021; Onagbiye et al., 2018; Kondo et al., 2023; El Fakir et al., 2014a; Olasehinde et al., 2024; Nkurunziza et al., 2016; Farid et al., 2023; Odetunde et al., 2020; Araya et al., 2019; Smith and Morris-Eyton, 2023; Westmoreland et al., 2018; Guermazi et al., 2012; Scott et al., 2017; Okello et al., 2018; Owolabi, 2010; Kulich et al., 2008; Gadisa et al., 2019), while 30.0% (*n* = 12) adopted interviews in data collection using 10 tools (Colbourn et al., 2012; Bowden et al., 2002; Namisango et al., 2007; Reba et al., 2019; Younsi and Chakroun, 2014; Ibrahim et al., 2020; Jikamo et al., 2021; Duracinsky et al., 2012; Borissov et al., 2022; El Fakir et al., 2014a; Osman et al., 2018; Ohrnberger et al., 2020), reflecting differences in participant literacy and accessibility. Validation efforts demonstrated robust reliability, with internal consistency (Cronbach’s alpha) exceeding 0.70 in the majority of the tools (see Table 3).

**Table 3:** Characteristics of the health-related quality of life tools.

S/no	Tools (authors)	Type	Language	Mode of administration	Domains of HRQoL	Scoring system	Development	Validation (yes/no)	Cultural adaptation
1.	WHOQOL-OLD (Van Biljon et al., 2015; Muhye and Fentahun, 2023)	Generic	English (Van Biljon et al., 2015) and Amharic (Muhye and Fentahun, 2023)	Self-administered (Van Biljon et al., 2015) and interview (Muhye and Fentahun, 2023)	Sensory abilities; autonomy; past, present, and future activities; social participation; death and dying; and intimacy	Each item is scored on a Likert-type scale ranging from 1 to 5, with higher scores representing greater QoL	No	Yes	No (Van Biljon et al., 2015) and yes (Muhye and Fentahun, 2023)
2.	STI-HRQoL long form 37 items (Smith and Morris-Eyton, 2023)	Specific	English	Self-administered and interview	Physical health, mental health, and socioeconomic health	Agreement scores on a scale of 0 to 1	Yes	Yes	No
3.	STI-HRQoL—short form 25 items (Smith and Morris-Eyton, 2023)	Specific	English	Self-administered and interview	Physical health, mental health, and socioeconomic health	Agreement scores on a scale of 0 to 1	Yes	Yes	No
4.	WHOQOL-BREF (Colbourn et al., 2012; Reba et al., 2019; Jikamo et al., 2021)	Generic	Amharic (Reba et al., 2019), Sidamic (Jikamo et al., 2021), and Chichewa (Colbourn et al., 2012)	Interview	Physical health, psychological health, social relationships, and environmental health	Each of these items was scored from 1 to 5 on a response scale, which is agreed as a 5-point Likert scale	No	Yes	Yes
5.	PROMIS-25 (Westmoreland et al., 2018)	Generic	Chichewa	Self-administered or proxy	Mobility, anxiety, depressive symptoms, fatigue, peer relationships, and pain interference	A 5-point Likert scale is used. Additionally, a single-item pain intensity measurement was scored from 0 to 10	No	Yes	Yes
6.	SF-12 (Younsi and Chakroun, 2014; Ibrahim et al., 2020; Ohrnberger et al., 2020)	Generic	Tunisian (Younsi and Chakroun, 2014), Hausa (Ibrahim et al., 2020), and Chi Chewa, Chi Yao or Chi Tumbuka (Ohrnberger et al., 2020)	Interview	Physical functioning, role limitation due to physical problems, bodily pain, general health, vitality, social functioning, role limitation due to emotional problems, perceived mental health	Response for raw scores for some items ranges from 1 to 6. The eight-scale scores range from 0 (the worst) to 100 (the best)	No	Yes	Yes
7.	SF-36 (Mbada et al., 2015; Guermazi et al., 2012; Okello et al., 2018)	Generic	Yoruba (Mbada et al., 2015), Tunisian Arabic (Guermazi et al., 2012), English (Mbada et al., 2015; Okello et al., 2018)	Self-administered (Guermazi et al., 2012) and interview (Okello et al., 2018)	Physical and mental health components with eight subscales: physical functioning, role limitations due to physical problems, bodily pain, general health, vitality, social functioning, role limitations due to emotional problems, and mental health	Standard SF-36 scoring (0–100 scale, higher scores indicate better health)	No	Yes	Yes
8.	MVQOLI (Namisango et al., 2007)	Generic	Luganda and English	Self-administered and interview	Symptoms, functional status, interpersonal relations, emotional wellbeing, and transcendence	A 5-point Likert scale is used, with domain scores and a total score formula; the lowest score indicates the least desirable situation and vice versa	No	Yes	Yes
9.	I-OPQOL (Mgbeojedo et al., 2022)	Generic	Igbo	Self-administered and interview	Life overall; health; social relationships; independence control; home and neighborhood; psychological and emotional wellbeing; financial circumstances; religion and culture	Each participant was expected to answer “YES” or “NO” for each item and response option	Yes	Yes	Yes
10.	EQ-5D-Y (Ravens-Sieberer et al., 2010; Scott et al., 2017)	Generic	Afrikaans (Scott et al., 2017) and English (Ravens-Sieberer et al., 2010)	Self-administered	Mobility, looking after myself, doing usual activities, pain or discomfort, and worried, sad, or unhappy	Each participant is required to fill in a visual analog scale (VAS), which ranges from 0, the worst health state imaginable, to 100, the best health state imaginable	No	Yes	NO
11.	EORTC QLQ-BR45 (Ehab et al., 2021; Kidayi et al., 2023; Getu et al., 2022)	Specific	Egyptian Arabic (Ehab et al., 2021), Swahili (Kidayi et al., 2023), and Amharic (Getu et al., 2022)	Interview (Ehab et al., 2021; Kidayi et al., 2023) and Self-administered (Getu et al., 2022)	The EORTC QLQ-BR45 comprises four functional scales (body image, sexual functioning, sexual enjoyment, and future perspective) and five symptom scales/items (systemic therapy side effects, breast symptoms, arm symptoms, and upset by hair loss)	These tools use a 4-point scale from 1 = not at all, to 4 = very much, and a scoring scale of 0–100, with a high score indicating better functioning and severity for high symptoms/item scale	No	Yes	Yes
12.	PROQOL-HIV (Duracinsky et al., 2012)	Specific	French	Interview	PROQOL-HIV has 11 themes: general health perception, social relationships, emotions, energy/fatigue, sleep, physical and daily activity, coping, future cognitive functioning, symptoms, and treatment. The remaining three non-HRQL items concerned satisfaction with HIV healthcare services, financial difficulties due to HIV, and concerns about having a child	PROQOL-HIV domain is on a 5-point scale ranging from 0—never to 4—always, except for one item whose response scale is 0—very good to 4—very poor. For the EQ-5D, the domain is on a 3-point scale and a 100-point visual analog scale (VAS), ranging from best to worst imaginable health state of self-perceived general health	No	Yes	Yes
13.	EORTC QLQPAN26 (Gqada et al., 2021)	Specific	isiXhosa and Afrikaans	Self-administered and interview	Seven multi-item symptom scales in the QLQ-PAN26, namely pancreatic pain, gastrointestinal symptoms, altered bowel habits, hepatic, body image, healthcare satisfaction, and sexuality	A 4-level Likert scale for the languages from previous translations was used	No	Yes	Yes
14.	SF-8 (Onagbiye et al., 2018)	Generic	South African English and Setswana	Self-administered	Physical functioning, role limitation due to physical problems, bodily pain, general health, vitality, social functioning, role limitation due to emotional problems, perceived mental health	The scoring in SF-8 was based on this two-component summary and was calculated by weighting each SF-8 item using a norm-based procedure in the instrument guidelines	No	Yes	Yes
15.	KCCQ (Okello et al., 2018)	Specific	Not stated	Self-administered	KCCQ has physical limitation, symptom stability, symptom frequency, symptom burden, self-efficacy, quality of life, and social limitation. The SF-36 questionnaire covers physical functioning, physical limitation, emotional limitation, bodily pain, general health, mental health, social functioning, energy fatigue, and physical health	Both were measured using a Likert scale, but the KCCQ was also measured with two summary subscales: the overall KCCQ score and the clinical summary score. The scores for both ranged from 0 to 100, with higher scores indicating better health status	No	Yes	No
16.	D39 (Uwizihiwe et al., 2022)	Specific	Kinyarwanda	Interviews	It consists of 39 items grouped into five dimensions: Energy and mobility, diabetes control, social burden, anxiety and worry, and sexual functioning	Each item can be answered using a 7-point scale ranging from 0.5 (not affected at all) to 7.5 (extremely affected)	No	Yes	Yes
17.	HRQOLISP (Owolabi, 2010)	Specific	English and German	Self-administered and interview	It comprises two spheres and seven domains. The physical sphere includes physical, psychological, cognitive, and ecosocial domains, whereas the spiritual sphere consists of soul, spirit, and spiritual interaction domains	The domain scores were transformed into a scale with a maximum score of 100 (best health) each	No	Yes	No
18.	EORTC QLQ-C30 (Kidayi et al., 2023; Araya et al., 2019; El Alami et al., 2021; Gadisa et al., 2019)	Specific	Swahili (Kidayi et al., 2023) Amharic (Araya et al., 2019; Gadisa et al., 2019) Moroccan Arabic (El Alami et al., 2021)	Interview (Kidayi et al., 2023; El Alami et al., 2021) and self-administered (Araya et al., 2019; El Alami et al., 2021)	It is sub-grouped into 15 domains, including five functional subscales (physical functioning, role functioning, emotional functioning, cognitive functioning, and social functioning); three multi-item symptom subscales (fatigue, nausea/vomiting, and pain); global health status/QoL subscale; and six single items addressing various symptoms and perceived financial impact	The item scoring procedure for the EORTC QLQ-C30 was managed according to the EORTC QLQ-C30 scoring manual. After the scoring procedures, the score was transformed into a 0–100 scale	No	Yes	Yes
19.	BREAST-Q questionnaire (Olasehinde et al., 2024)	Specific	Yoruba	Self-administered	Quality of Life (including psychosocial wellbeing, sexual wellbeing, physical wellbeing of the chest, and adverse effects of radiation) and Satisfaction (including satisfaction with breasts, surgeons, the medical team, and office staff). The psychosocial wellbeing, sexual wellbeing, physical wellbeing of the chest, and satisfaction with breast domains are applicable in the preoperative setting, while all the domains can be used postoperatively	BREAST-Q scores are transformed onto a scale from 0 to 100	No	Yes	Yes
20.	SF-NDI (Nkurunziza et al., 2016)	Specific	Kinyarwanda	Self-administered	The SF-NDI evaluates tension/ anxiety, interference with daily activities, disruption of usual eating/drinking, knowledge of/control over disease symptoms, and interference with work/study with 2-item 5-point Likert scales, with a total score calculated as the mean of the five subscale scores	Each item is assigned a numerical score that is summed into a total score; scores >14	No	Yes	Yes
21.	Modified pTESS (Farid et al., 2023)	Specific	Arabic	Self-administered	Modified versions of pTESS included the same additional mental domain, which involved six questions that were adopted from the pediatric anger, fatigue, cognitive, and depression domains of the NeuroQOL system, as well as the mental component of SF-36	5-point Likert scale	No	Yes	Yes
22.	Modified TESS (Farid et al., 2023)	Specific	Arabic	Self-administered	Modified versions of TESS included the same additional mental domain, which involved six questions that were adopted from the pediatric anger, fatigue, cognitive, and depression domains of the NeuroQOL system, as well as the mental component of SF-36	5-point Likert scale	No	Yes	Yes
23.	EQ-5D-3L (Duracinsky et al., 2012; El Fakir et al., 2014b)	Generic	Moroccan Arabic (El Fakir et al., 2014b), English (Duracinsky et al., 2012)	Interview	Mobility, self-care, usual activities, pain/discomfort, and anxiety/depression	Scores for the emotional, functioning, and symptom scales are expressed in a linear scale, varying from 0 (no effect on QoL) to 100 (maximum effect on QoL)	No	Yes	Yes
24.	Skindex-16 (El Fakir et al., 2014b)	Specific	Moroccan Arabic	Interview	It is composed of 16 items grouped under three components: symptoms (four items; nos. 1–4), emotions (seven items; nos. 5–11), and functioning (five items; nos. 12–16)	Scores for the emotional, functioning, and symptom scales are expressed in a linear scale, varying from 0 (no effect on QoL) to 100 (maximum effect on QoL)	No	Yes	Yes
25.	SS-QoL 2.0 (Odetunde et al., 2020)	Specific	Igbo	Self-administered	Cognitive	Descriptive statistics of mean and standard deviation were used to analyze domains and overall scores on SS-QoL	No	Yes	Yes
26.	EORTC QLQ-CX24 (Araya et al., 2019)	Specific	Amharic	Self-administered	Physical functioning, role functioning, emotional functioning, cognitive functioning, and social functioning, three multi-item symptom subscales (fatigue, nausea/vomiting, and pain); global health status/QoL subscale; and six single items addressing various symptoms and perceived financial impact, body image domain, and four items with the sexual/vaginal functioning domain	The standard scoring algorithm recommended by the EORTC was used to linearly transform all scales and item scores to a 0–100 scale	No	Yes	Yes
27.	Quality of Life in Reflux and Dyspepsia (QOLRAD) (Kulich et al., 2008)	Specific	Afrikaans	Self-administered	Five dimensions: emotional distress, sleep disturbance, vitality, food/drink problems, and physical/social functioning	The questions are rated on a 7-point graded Likert scale; lower values indicate a more severe impact on daily functioning	No	Yes	Yes
28.	Prolapse quality-of-life questionnaire (P-QoL) (Brandt et al., 2016)	Specific	Afrikaans		P-QOL domain includes general health, prolapse impact, role limitations, physical limitations, social limitations, personal relationships, emotional problems, sleep/energy disturbances, and prolapse severity	All asymptomatic participants were stage 0 on the POP-Q system, and the symptomatic participants were at stages III and IV	No	Yes	Yes
29.	PedsQL™ FIM (acute version) (Borissov et al., 2022; Farid et al., 2023)	Generic	Ethiopia	Interview	The scale comprises 36 items across eight subscales: physical functioning (6 items), emotional functioning (5 items), social functioning (4 items), cognitive functioning (5 items), communication (3 items), worry (5 items), daily activities (3 items), and family relationships (5 items)	PedsQL™ FIM total score, all items are reverse-coded and rescaled to 0, 25, 50, 75, and 100	No	Yes	Yes
30.	WHOQOL-HIV BREF questionnaire (Kondo et al., 2023)	Specific	Kiswahili	Self-administered	Physical, psychological, level of independence, social relationships, environment, and spirituality/religion/personal beliefs domains	Each domain has different facets, which were rated on a 5-point Likert scale, where 1 indicated a negative perception and 5 indicated a positive perception. The original English WHOQOL-HIV BREF questionnaire was used to assist with scoring and coding	No	Yes	Yes
31.	EORTC QLQ-BR-23 (El Fakir et al., 2014a; Gadisa et al., 2019)	Specific	Moroccan Arabic (El Fakir et al., 2014a) Amharic (Gadisa et al., 2019)	Self-administered	Two multi-item functional scales (body image and sexual functioning), three symptom scales (systemic side effects, breast symptoms, and arm symptoms), and three single-item scales on sexual enjoyment, future perspectives, and upset by hair loss	Each item was scored on a 4-point Likert scale [“not at all” (WHO, 2020a), to “very much” (Tran et al., 2012)], and the time frame was “during the past week,” except for the sexual items (“during the past 4 weeks”). In summary, 0–100 scale	No	Yes	Yes
32.	GOHAI (Osman et al., 2018)	Specific	Arabic	Interview	Satisfaction with retention, comfort, stability, ability to speak, and overall satisfaction with maxillary and mandibular complete dentures	“Satisfied,” “regular,” or “dissatisfied” scores of 2, 1, or 0	No	Yes	Yes
33.	OHIP-14 (Osman et al., 2018)	Specific	Arabic	Interview	Satisfaction with retention, comfort, stability, ability to speak, and overall satisfaction with maxillary and mandibular complete dentures	“Satisfied,” “regular,” or “dissatisfied” scores of 2, 1, or 0	No	Yes	Yes
34.	KENQOL (Bowden et al., 2002)	Generic	Kikamba	Interview	Positive and negative aspects of health that comprised “contentment,” “cleanliness,” “corporeal capacity,” “co-operation,” and “completeness”		Yes	Yes	Yes

WHOQOL-OLD, World Health Organization quality of life for older adults; STI-HRQoL, Smith Toolkit for Integrated Health-Related Quality of Life; WHOQOL-BREF, World Health Organization quality of life (brief version); PROMIS, Patient-Reported Outcomes Measurement Information System; SF-12, 12-item short-form health survey; SF-36, 36-item short-form health survey; MVQOLI, Missoula-Vitas Quality of-Life Index; I-OPQOL-35, Igbo version of Older People’s Quality of Life Questionnaire (35 items). EQ-5D-Y, EuroQol Group 5-dimension for children and adolescents; EORTC QLQ-BR45, European Organization for Research and Treatment of Cancer—quality of life questionnaire for breast cancer patients (45 items); PROQOL-HIV, Patient Reported Outcomes Quality of Life–HIV questionnaire; EORTC QLQ-PAN26, European Organization for Research and Treatment of Cancer—quality of life questionnaire for patients with pancreatic cancer and chronic pancreatitis (26 items); SF-8, 8-item short-form survey; KCCQ, Kansas City Cardiomyopathy Questionnaire. D-39, diabetes (39 items); HRQOLISP-40, health-related quality of life in stroke patients (40 items); EORTC QLQ-C30, European Organization for Research and Treatment of Cancer—quality of life questionnaire for colorectal cancer patients (30 items); BREAST-Q, Breast Questionnaire; SF-NDI, Short-Form Nepean Dyspepsia Index; pTESS, Toronto Extremity Salvage Score; TESS, Toronto Extremity Salvage Score. EQ-5D-3L, EuroQol Group 5-dimension, 3-level instrument; Skindex-16, the 16-item skin disease quality of life tool; SS-QoL, stroke-specific quality of life scale; QOLRAD, Quality of Life in Reflux and Dyspepsia; P-QOL, prolapse quality-of-life questionnaire; PedsQL™ FIM, Pediatric Quality of Life Inventory™ Family Impact Module; POP-Q, Pelvic Organ Prolapse Quantification. WHOQOL-HIV BREF, World Health Organization quality of life questionnaire for HIV patients; EORTC QLQ-BR23, European Organization for Research and Treatment of Cancer—quality of life questionnaire for breast cancer patients (23 items); GOHAI, Geriatric Oral Health Assessment Index; OHIP-14, Oral Health Impact Profile-14; KENQOL, Kenyan quality of life.

### Strengths and limitations of HRQoL tools

Many of the HRQoL tools demonstrated strong contextual adaptability, with WHOQOL-BREF, SF-36, and EQ-5D-Y (Colbourn et al., 2012; Reba et al., 2019; Jikamo et al., 2021; Mbada et al., 2015; Ravens-Sieberer et al., 2010; Guermazi et al., 2012; Scott et al., 2017; Kulich et al., 2008) validated across multiple African languages and populations, ensuring relevance in diverse settings. Disease-specific instruments such as the EORTC QLQ-BR45 (Ehab et al., 2021; Kidayi et al., 2023; Getu et al., 2022) for breast cancer and the KCCQ (Okello et al., 2018) for heart failure provided highly specialized assessments, capturing condition-specific impacts more accurately than generic tools. Nonetheless, some instruments, such as the D-39 for diabetes (Uwizihiwe et al., 2022) and the EORTC QLQ-PAN26 for pancreatic cancer (Gqada et al., 2021), were lengthy and complex, posing challenges for use in clinical settings with time-constrained patients (see Table 4).

**Table 4:** Strengths and limitations of the health-related quality of life tools.

S/no	Tools	Strength	Limitations
1.	WHOQOL-OLD (Van Biljon et al., 2015; Muhye and Fentahun, 2023)	The WHOQOL-OLD can be used for a broad population; it has domains specific to the older population, and being translated to the Amharic version shows it can be contextual to a particular population based on their local language	It lacks disease-specific sensitivity when compared to other HRQoL tools like EQ-5D; it may be too long for older populations to answer all the questions
2.	STI-HRQoL long form 37 items (Smith and Morris-Eyton, 2023)	STI-HRQoL was developed to assess non-communicable diseases [hypertension, type 2 diabetes, and cardiovascular disease (CVD)]. It has the domain that measures physical health, mental health, and socioeconomic health dimensions	It cannot be used for communicable diseases. It is too long to fill
3.	STI-HRQoL short form 25 items (Smith and Morris-Eyton, 2023)	STI-HRQoL was developed to assess non-communicable diseases [hypertension, type 2 diabetes, and cardiovascular disease (CVD)]. It has the domain that measures physical health, mental health, and socioeconomic health dimensions. It is shorter than the 37-item STI-HRQoL	It cannot be used for communicable diseases. It is too long to fill
4.	WHOQOL-BREF (Colbourn et al., 2012; Reba et al., 2019; Jikamo et al., 2021)	These tools were translated into Amharic, Sidamic, and Chichewa, making them contextually relevant for that population. It is ideal for general life assessment. It contains 26 items, which makes it longer than the WHOQOL-100	It has limited disease-specific sensitivity. It is less detailed
5.	PROMIS-25 (Westmoreland et al., 2018)	PROMIS is more valid, reliable, and responsive than other PRO instruments. The PROMIS instruments can be used for general disease states and administered across several languages and cultural contexts. Its translation to Chichewa made it easier to assess the HRQoL of pediatric patients with lymphoma in Malawi	It is not disease-specific. It does not have emphasis on the environmental factors like the WHOQoL
6.	SF-12 (Younsi and Chakroun, 2014; Ibrahim et al., 2020; Ohrnberger et al., 2020)	The SF-12 is one of the most widely used and well-validated HRQoL. The SF-12 short-form HRQoL is shorter than the SF-36. It has been used in various populations, which proves its strong contextual property	The SF-12 short-form HRQoL is less detailed than the SF-36. It has a complex scoring system
7.	SF-36 (Mbada et al., 2015; Guermazi et al., 2012; Okello et al., 2018)	The SF-36 is one of the most widely used and well-validated HRQoL. It has been used in various populations which proves its strong contextual property	The SF-36 is the longest of the short-form HRQoL, and it has a complex scoring system
8.	MVQOLI (Namisango et al., 2007)	MVQOLI is a tool that is used in advanced illness and palliative care settings. MVQOLI is preferred over other tools because it measures the transcendence/existential domain. It also showed	It is not suitable for the general population. Its scoring system is complex
9.	I-OPQOL (Mgbeojedo et al., 2022)	OPQOL is used in determining the quality of life of the older population. The I-OPQOL covers the population that has a low English literacy level. The OPQOL was translated into different languages, showing a contextual property	It cannot be used for pediatric or adolescent population
10.	EQ-5D-Y (Ravens-Sieberer et al., 2010; Scott et al., 2017)	The EQ-5D-Y is one of the most widely used and well-validated HRQoL instruments in the adolescent population. It has been used in various populations, which proves its strong contextual property. It is shorter than the SF versions	It is not specific to a disease
11.	EORTC QLQ-BR45 (Ehab et al., 2021; Kidayi et al., 2023; Getu et al., 2022)	EORTC QLQ-BR45 can assess more accurately and comprehensively the impact of new treatments on breast cancer patients’ QoL. It has been validated in several countries	It is not suitable for other forms cancers. It takes longer time to complete
12.	PROQOL-HIV (Duracinsky et al., 2012)	PROQOL-HIV is tailored for HIV management, and it is practical and easier to administered	It is specific to HIV
13.	EORTC QLQPAN26 (Gqada et al., 2021)	EORTC QLQPAN26 incorporates several symptom scales relevant to Pancreatic ductal adenocarcinoma and comprises 26 questions that address unique symptoms and treatments	It is only applicable to pancreatic cancer patients. It is long and not focused on comorbidity
14.	SF-8 (Onagbiye et al., 2018)	The SF-8 is one of the most widely used and well-validated HRQoL. The SF-8 short-form HRQoL is shorter than the SF-12 and SF-36. It has been used in various populations, which proves its strong contextual property	The SF-8 short-form HRQoL is less detailed than the SF-12 and SF-36. It has a complex scoring system
15.	KCCQ (Okello et al., 2018)	KCCQ is the most widely used HRQoL for heart failure patients. It has been culturally adapted and translated	It is specific and focuses on symptom reporting
16.	D39 (Uwizihiwe et al., 2022)	The Diabetes-39 (D-39) questionnaire is a widely used self-reporting tool; it measures glycaemic control, adherence to treatment, and complications and has been linked to other associated constructs of QoL	It is not applicable to other forms of disease, and it is too long
17.	HRQOLISP (Owolabi, 2010)	HRQOLISP measures the spiritual spheres of the quality of life of the patients. It was specific to stroke patients. For this study, it was shortened from 104 to 40, and it was contextual	It is disease-specific and cannot be used for other health conditions. Although shortened, it was still too long to be used
18.	EORTC QLQ-C30 (Kidayi et al., 2023; Araya et al., 2019; El Alami et al., 2021; Gadisa et al., 2019)	It is a psychometrically robust, cross-culturally accepted, and most frequently used tool to assess HRQoL in cancer patients. It is also contextual	Specific for cancer patients and long-term
19.	BREAST-Q questionnaire (Olasehinde et al., 2024)	This tool measures the QoL and satisfaction of patients following breast surgery. It has been translated into 30 languages globally	It is used just to assess the QoL and satisfaction after the surgery has been done, not particularly for living with the disease condition
20.	SF-NDI (Nkurunziza et al., 2016)	It is specific to patients living with dyspepsia. It also proves to be contextual since it was translated into the local language in Rwanda	It is disease-specific and hence cannot be used for other disease conditions
21.	Modified pTESS (Farid et al., 2023)	It was developed for ages ranging from 6 to 17.5 years. It was able to be used to determine the HRQoL of children and adolescents	It cannot be used for adults and is specific to children with bone sarcoma
22.	Modified TESS (Farid et al., 2023)	TESS was originally developed for an age group ranging from 12 to 60 years, and it was modified to measure the mental domain. It has been culturally adapted in several countries	It includes items that seem irrelevant for children and adolescents and are specific to the population with bone cancer
23.	EQ-5D-3L (Duracinsky et al., 2012; El Fakir et al., 2014b)	The EQ-5D-Y is one of the most widely used and well-validated HRQoL for the entire population. It has been used in various populations, which proves its strong contextual property. It is shorter than the SF versions	It is not specific to a disease
24.	Skindex-16 (El Fakir et al., 2014b)	It was used to measure burden symptoms, physical and emotional state related to skin disease. It is specific to only skin disease	It cannot be used for other disease conditions
25.	SS-QoL 2.0 (Odetunde et al., 2020)	It is a comprehensive tool to measure the multiple impacts of stroke. It is contextual for the Nigerian population	It can only be used for patients who have suffered from a stroke. It is also disease-specific
26.	EORTC QLQ-CX24 (Araya et al., 2019)	EORTC QLQ-CX24 measures the QoL of cervical cancer patients, and it was made contextual to the Ethiopian population	It is disease-specific and uses a standard algorithm from EORTC for scoring
27.	Quality of Life in Reflux and Dyspepsia (QOLRAD) (Kulich et al., 2008)	It is specific to patients living with GERD. It also proves to be contextual since it was translated into the local language in South Africa	It is disease-specific and cannot be used for other disease conditions
28.	Prolapse quality-of-life questionnaire (P-QoL) (Brandt et al., 2016)	P-QoL is the only reliable tool to measure QOL of urogenital prolapse. It has been translated into eight languages, which shows its contextual power	It is disease-specific and used only for women
29.	PedsQL™ FIM (acute version) (Borissov et al., 2022; Farid et al., 2023)	PedsQL™ FIM provides insight into the impact of the child’s condition on the caregiver	It measures the quality of life of the caregiver and not the quality of life of the child suffering from the disease condition. It is subjective
30.	WHOQOL-HIV BREF questionnaire (Kondo et al., 2023)	WHOQOL-HIV BREF is tailored for HIV management, and it is practical and easier to administer. It is short compared to the other versions of WHOQOL	It is not generic like other WHOQOL
31.	EORTC QLQ-BR-23 (El Fakir et al., 2014a; Gadisa et al., 2019)	EORTC QLQ-BR23 measures the QoL of breast cancer patients, and it was made contextual to the Moroccan population. It is shorter than the EORTC QLQ-BR45	It is not suitable for other forms of cancer
32.	GOHAI (Osman et al., 2018)	An oral HRQoL specifically for patients who have recently undergone denture therapy for the first time	It was not used for patients who have had recurrent denture therapy. It is specific to oral health alone
33.	OHIP-14 (Osman et al., 2018)	An oral HRQoL specifically for patients who have recently undergone denture therapy for the first time	It was not used for patients who have had recurrent denture therapy. It is specific to oral health alone
34.	KENQOL (Bowden et al., 2002)	A thorough validation process to produce the only locally developed HRQoL in Africa	It was not used for any disease condition, and the use of a local language restricts its adaptability to other regions

### Overview of the findings, strengths, and limitations of the included studies in the review

Reliability and validity were good in 87.5% (*n* = 35) of the tools in African contexts (Colbourn et al., 2012; Namisango et al., 2007; Van Biljon et al., 2015; Reba et al., 2019; Younsi and Chakroun, 2014; Ibrahim et al., 2020; Jikamo et al., 2021; Mbada et al., 2015; Mgbeojedo et al., 2022; Muhye and Fentahun, 2023; Ravens-Sieberer et al., 2010; Ehab et al., 2021; Duracinsky et al., 2012; Onagbiye et al., 2018; Uwizihiwe et al., 2022; Kidayi et al., 2023; Brandt et al., 2016; Borissov et al., 2022; Kondo et al., 2023; El Fakir et al., 2014a; Olasehinde et al., 2024; Nkurunziza et al., 2016; Farid et al., 2023; El Fakir et al., 2014b; Odetunde et al., 2020; Smith and Morris-Eyton, 2023; Westmoreland et al., 2018; Guermazi et al., 2012; Scott et al., 2017; Ohrnberger et al., 2020; Okello et al., 2018; Owolabi, 2010; Kulich et al., 2008; Getu et al., 2022; Gqada et al., 2021). Cultural adaptation was found to enhance the usability and acceptability of tools in 75.0% (*n* = 30) of the studies. Generic tools, such as the WHOQOL-BREF and SF-12, were validated across diverse populations and conditions, constituting 15.0% (*n* = 6) of the studies, highlighting their versatility (Colbourn et al., 2012; Reba et al., 2019; Younsi and Chakroun, 2014; Ibrahim et al., 2020; Jikamo et al., 2021; Ohrnberger et al., 2020). However, 60.0% (*n* = 24) of studies reported limitations related to sample size, underscoring the need for larger, more representative cohorts to improve generalisability (Colbourn et al., 2012; Namisango et al., 2007; Van Biljon et al., 2015; Reba et al., 2019; Jikamo et al., 2021; Mgbeojedo et al., 2022; Muhye and Fentahun, 2023; Ehab et al., 2021; Duracinsky et al., 2012; Onagbiye et al., 2018; Uwizihiwe et al., 2022; Kidayi et al., 2023; Brandt et al., 2016; Borissov et al., 2022; Olasehinde et al., 2024; Farid et al., 2023; Odetunde et al., 2020; El Alami et al., 2021; Osman et al., 2018; Westmoreland et al., 2018; Guermazi et al., 2012; Scott et al., 2017; Owolabi, 2010; Gadisa et al., 2019). Policy implications from 80.0% (*n* = 32) of the studies emphasized the integration of HRQoL tools into healthcare systems to facilitate patient-centered care and inform resource allocation strategies (Colbourn et al., 2012; Bowden et al., 2002; Namisango et al., 2007; Van Biljon et al., 2015; Reba et al., 2019; Younsi and Chakroun, 2014; Ibrahim et al., 2020; Jikamo et al., 2021; Mbada et al., 2015; Mgbeojedo et al., 2022; Muhye and Fentahun, 2023; Ravens-Sieberer et al., 2010; Ehab et al., 2021; Duracinsky et al., 2012; Gqada et al., 2021; Onagbiye et al., 2018; Uwizihiwe et al., 2022; Kidayi et al., 2023; Brandt et al., 2016; Borissov et al., 2022; Kondo et al., 2023; El Fakir et al., 2014a; Nkurunziza et al., 2016; Farid et al., 2023; Smith and Morris-Eyton, 2023; Westmoreland et al., 2018; Guermazi et al., 2012; Scott et al., 2017; Ohrnberger et al., 2020; Okello et al., 2018; Kulich et al., 2008; Getu et al., 2022) (see Table 5).

**Table 5:** Overview of the findings, strengths, and limitations of the included studies in the review.

Ref. no.	Key findings	Policy implications	Strengths	Limitations
1.	Encouraging results related to the original factor structure (α > 0.80) of the WHOQOL-OLD and the other three short versions of this instrument used	Understanding QoL in older adults can inform policy and care decisions	WHOQOL-OLD is recommended for comprehensive QoL assessment	Findings cannot be generalized due to cultural and sample size limitations
2.	Both forms of the toolkit (STI-HRQoL) were highly reliable (Pearson’s *r* = 0.89*; 0.89*, Spearman’s rho = 0.88*; 0.89*, ICC = 0.94*; 0.94*) and can be used as a cost-effective tool to assess and manage non-communicable diseases	Healthcare professionals can better assess QoL in patients with non-communicable diseases	The toolkit is cost-effective and comprehensive	Cross-sectional design limits causality determination
3.	The Amharic version of the WHOQOL-BREF instrument has internal consistency and validity (α ≥ 0.7) to investigate quality of life among patients with diagnosed type 2 diabetes	Policymakers can improve QoL for diabetic patients	English version translated into Amharic and tested on patients	Conducted at a single referral center, limiting generalisability
4.	Translation and cultural validation of the PROMIS-25 into Chichewa for Malawi was successfully carried out (*α* = 0.71–0.93)	The questionnaire can be used in future research and practice	First study to translate a pediatric HRQoL tool into a Bantu language	No cross-cultural validation or test–retest reliability assessed
5.	Tunisian SF-12 showed satisfactory internal consistency and convergent validity. Cronbach’s α coefficient for the physical component summary (PCS) score and mental component summary (MCS) score was 0.73 and 0.72, respectively	The tool can be used in research and clinical practice	Tunisian SF-12 is reliable and valid for HRQoL measurement	Cross-sectional design limits causality assessment
6.	The Hausa version of SF-12 can be used clinically and for research in Hausa-speaking patients with chronic LBP. The physical component summary and the mental component summary showed acceptable (α = 0.69 and 0.79, respectively)	The instrument can be deployed for clinical and research purposes in Hausa-speaking patients with chronic LBP	This is the first study to examine the factorial or measurement invariance of the SF-12 in the population	Some steps in the forward and backward translations were skipped
7.	Although the culturally adapted WHOQOL-BREF had internal consistency reliability ranging from 0.8045 to 0.9123, indicating good-to-excellent reliability, it still failed the assumptions because it did not measure the same concepts in the original and target settings	Findings can improve QoL assessment in clinical settings	Conceptual equivalence and qualified translators enhanced tool quality	Cross-sectional design and recall bias are limitations
8.	Yoruba SF-36 demonstrated excellent psychometric properties with ranges between 0.749 and 0.902	The Yoruba version simplifies QoL assessment for less literate populations	High response rate and comprehensive adaptation process	Single cultural focus and sampling heterogeneity
9.	MVQOLI-M showed high reliability for AIDS patients in palliative care. The instrument demonstrated good internal consistency (*α* = 0.83)	Supports holistic QoL assessment in low-resource settings	First validation of MVQOLI in an African setting	Potential cultural bias and limited generalisability
10.	Chichewa WHOQOL-BREF showed acceptable internal consistency (α > 0.7) except for the social domain	Validated tool can be integrated into healthcare systems	High response rate and diverse sample population	Potential sample bias and lack of test–retest reliability
11.	Tunisian Arabic SF-36 is reliable and valid for HRQoL assessment, with *α* = 0.94	The tool aids public health research and planning	High reliability and cultural adaptation to Tunisia	Illiteracy limits tool use; sample not fully representative
12.	I-OPQOL showed excellent concurrent validity and good reliability, *α* = 0.78	Tool recommended for translation into other Nigerian languages	Translation process involved multiple translators	Small sample size and exclusion of non-English speakers
13.	Amharic WHOQOL-OLD showed high reliability for older adults in Ethiopia, with α = 0.96	Tool can be used by social care organizations for policy impact	Data collection involved experienced health workers	Limited to urban settings; no test–retest reliability assessed
14.	EQ-5D-Y performed well in acutely ill children but not in other groups. The dimensions were reliable because the kappa varied from 0.365 to 0.653	The tool is short, responsive, and acceptable for acute care	Useful information yielded for routine use	Small sample size in chronically ill groups may bias results
15.	EQ-5D-Y is feasible, reliable, and valid for South African children (kappa coefficients were up to 0.67)	Helps policymakers improve QoL for children and adolescents	Multinational scope identified significant group differences	No specific population performance data due to ethical constraints
16.	EORTC QLQ-BR45 showed good reliability except for the body image scale (α ≥ 0.7)	Effective tool for assessing HRQoL in breast cancer patients	Cross-cultural adaptation and validation in Egypt	Low education level led to interviewing patients, limiting responses
17.	PROQOL-HIV is a valid and reliable instrument (*α* = 0.77–0.89) for assessing HRQL specific to HIV disease in different cultures and healthcare systems, showing a significant impact on the stigma scale in Senegal	Appropriate for international studies and clinical trials	Covers previously neglected dimensions like lipodystrophy	Small sample size and cross-sectional design
18	IsiXhosa and Afrikaans versions of EORTC QLQ-PAN26 showed high reliability for PDAC patients. For isiXhosa (α = 0.88) and Afrikaans (*α* = 0.89)	Effective HRQoL assessment for pancreatic cancer patients	Translation makes the tool understandable for the South African population	Religious and healthcare insurance factors may affect responses
19.	Setswana SF-8 is valid and feasible for HRQoL assessment in South Africa, α = 0.86	Setswana version simplifies HRQoL assessment in the Northwest province	First translation of SF-8 into Setswana for South Africa	Small sample size and a limited acceptability study
20.	Malawian SF-12 showed differences in the mental health construct compared to the US (*α* > 0.7)	Malawian SF-12 should be used for future analyses	First study to compute SF-12 health dimension weights in sub-Saharan Africa	Construct validity tested on a different sample
21.	KCCQ predicted mortality in heart failure patients within 3 months. The internal consistency of the overall KCCQ scale was excellent (*α* = 0.87)	KCCQ can predict mortality in acute heart failure patients	Longitudinal study with daily follow-up during hospitalization	Self-reported data may introduce reporting bias
22.	Kinyarwanda D-39 is reliable and valid for diabetic patients in Rwanda, with a composite reliability of above 0.7	Provides insights into QoL factors in the Rwandan cultural context	First tool tailored for Rwandan and sub-Saharan African contexts	Small sample size and disease variability may affect QoL
23.	HRQOLISP-40 showed good internal consistency (*α* > 0.7) in multicultural settings	Proposed for routine use and clinical trials in stroke patients	First stroke-specific HRQoL measure developed in Africa and Europe	Limited neuroimaging data in Nigeria compared to Germany
24.	Swahili EORTC QLQ-BR45 is reliable and valid for breast cancer patients (*α* > 0.7)	Simplifies data collection and improves QoL assessment in the region	Sample size considered representative of Tanzania	Low response rate for sexual functioning, limited evaluation
25.	Afrikaans P-QoL is reliable and feasible for urogenital prolapse management, *α* = 0.94, and good strength of agreement between items (*к* = 0.41–0.80)	Can be effectively used in South African women	Similar findings to previous validation studies strengthen results	Limited population size raises concerns about content validity interpretation
26.	Afrikaans QOLRAD is reliable for use in clinical trials, *α* = 0.79–0.95	Helps physicians interpret clinical benefits for patients	Enables comparisons between various countries	Paper versions may affect results compared to electronic data collection
27.	ATEC and PedsQL™ FIM are reliable for neurodevelopmental disorders in Ethiopia (*α* = 0.8–0.89 and ⩾0.9)	Advances in research on neurodevelopmental disorders in LMICs	First validation of PedsQL™ FIM in caregivers of children with disorders	Limited to help-seeking families in Addis Ababa; no autism severity measures
28.	WHOQOL-HIV BREF is reliable and valid for Tanzanian PLWHA (*α* = 0.89–0.90)	Supports resource allocation and intervention planning for PLWHA	Strong construct validity evidence supports its use in QoL screening	Cross-sectional design limits the sensitivity to change assessment
29.	Moroccan Breast Cancer-Specific Quality of Life Questionnaire QLQ-BR23 is reliable and valid for breast cancer patients (*α* > 0.7)	Useful in clinical trials evaluating interventions on QoL	First validation of QLQ-BR23 in Morocco	Responsiveness over time was not assessed
30.	Amharic EORTC QLQ-BR45 is reliable and valid for breast cancer patients in Ethiopia, *α* = 0.80	Further studies on responsiveness and test–retest analysis are recommended	First validation study on the updated QLQ-BR45 tool	No comparison studies available; responsiveness not assessed
31.	Yoruba BREAST-Q mastectomy module captures experiences of Nigerian women. Internal consistency *α* > 0.70	Provides a basis for contextually relevant interventions	Successful translation into Yoruba, a major Nigerian language	Unknown if women stabilize in all health domains within the study timeframe
32.	Kinyarwanda SF-LDQ and SF-NDI are reliable for dyspepsia patients in Rwanda with *α* = 0.93 and *α* = 0.92, respectively	Recommended for clinical and research initiatives in sub-Saharan Africa	Concurrent measurement of dyspepsia severity and QoL impact	No gold standard comparison: survey methods differed between time points
33.	Modified pTESS and TESS are valid for Egyptian childhood bone sarcoma patients with *α* > 0.9 for all the versions	Useful for initial assessment, treatment planning, and outcome evaluation	First study to modify and validate pTESS/TESS with a mental domain	Small sample size and cross-sectional design limit change detection
34.	Moroccan Arabic Skindex-16 is reliable for skin disease patients with *α* > 0.7	Measures the impact of skin disease on QoL in Moroccan patients	First validation of Skindex-16 in Arabic for Morocco	Further validation needed in regions with other languages
35.	Igbo SS-QoL 2.0 is valid and reliable for stroke survivors in Nigeria, *α* = 0.69–0.87	Recommended for assessing HRQoL among Igbo stroke survivors	Limited alterations from the original version with good validity	Generalisability limited to urban and semi-urban communities
36.	Amharic EORTC QLQ-CX24 is valid for cervical cancer patients in Ethiopia, *α* = 0.70–0.84	Useful in clinical and epidemiological cancer research	First study to validate EORTC QLQ-CX24 in Amharic	No test–retest reliability or responsiveness assessed
37.	Moroccan EORTC QLQ-C30 is reliable for colorectal cancer patients, α = 0.74	EORTC QLQ-C29 requires further exploration for clinical use	Provides valuable data for comparing HRQoL in colorectal cancer patients	Small sample size and potential shared variance in item-scale correlation
38.	Amharic EORTC QLQ-C30 and QLQ-BR23 are reliable for breast cancer patients in Ethiopia, *α* > 0.70 for all scales	Useful in research and clinical settings for HRQoL assessment	First validation of EORTC QLQ-C30 and QLQ-BR23 in Amharic	Test–retest reliability not measured
39.	OHIP-14 and GOHAI showed better OHRQoL in patients without denture experience, with a Cronbach alpha, *α* = 0.638 to 0.718	New measures like OHIP-EDENT may detect smaller changes in OHRQoL	Assessed the impact of complete denture therapy on OHRQoL in Sudan	Small sample size limited to Khartoum State; no clinical evaluation of dentures
40.	KENQOL was developed as a local HRQoL tool that provided valuable comparative data for explaining any similarities and dissonances in measured health in Kenya	There is a cultural perspective in the conceptualization of health, and this should be considered in QoL assessments	The qualitative methods used are recognized as participatory or rapid rural appraisal approaches in terms of the validity, reliability, and sensitivity of the instrument	The process was laborious and time-consuming. The authors acknowledged that other methods may be more appropriate for other research groups

### Summary of HRQoL tools following the consensus-based standards for the selection of health measurement instruments (COSMIN) guideline

A total of 34 HRQoL measurement tools were evaluated using the COSMIN framework. All the tools reported on feasibility, 33 (97.1%) tools assessed internal consistency using Cronbach’s alpha (Colbourn et al., 2012; Namisango et al., 2007; Van Biljon et al., 2015; Reba et al., 2019; Younsi and Chakroun, 2014; Ibrahim et al., 2020; Jikamo et al., 2021; Mbada et al., 2015; Mgbeojedo et al., 2022; Ravens-Sieberer et al., 2010; Ehab et al., 2021; Duracinsky et al., 2012; Onagbiye et al., 2018; Uwizihiwe et al., 2022; Kidayi et al., 2023; Kondo et al., 2023; El Fakir et al., 2014a; Olasehinde et al., 2024; Nkurunziza et al., 2016; Farid et al., 2023; Araya et al., 2019; El Alami et al., 2021; Osman et al., 2018; Smith and Morris-Eyton, 2023; Westmoreland et al., 2018; Guermazi et al., 2012; Scott et al., 2017; Ohrnberger et al., 2020; Okello et al., 2018; Owolabi, 2010; Kulich et al., 2008; Getu et al., 2022; Gadisa et al., 2019; Gqada et al., 2021). Construct validity was evaluated in 32 (94.1%) tools using methods such as confirmatory factor analysis (CFA) and assessments of convergent and discriminant validity (Colbourn et al., 2012; Namisango et al., 2007; Van Biljon et al., 2015; Reba et al., 2019; Younsi and Chakroun, 2014; Ibrahim et al., 2020; Jikamo et al., 2021; Mbada et al., 2015; Mgbeojedo et al., 2022; Ravens-Sieberer et al., 2010; Ehab et al., 2021; Duracinsky et al., 2012; Onagbiye et al., 2018; Uwizihiwe et al., 2022; Kidayi et al., 2023; Kondo et al., 2023; El Fakir et al., 2014a; Olasehinde et al., 2024; Nkurunziza et al., 2016; Farid et al., 2023; Odetunde et al., 2020; Araya et al., 2019; El Alami et al., 2021; Osman et al., 2018; Smith and Morris-Eyton, 2023; Westmoreland et al., 2018; Guermazi et al., 2012; Scott et al., 2017; Ohrnberger et al., 2020; Okello et al., 2018; Owolabi, 2010; Kulich et al., 2008; Getu et al., 2022; Gadisa et al., 2019; Gqada et al., 2021). Content validity, which assessed the relevance and comprehensiveness of the tools (Colbourn et al., 2012; Namisango et al., 2007; Van Biljon et al., 2015; Reba et al., 2019; Younsi and Chakroun, 2014; Ibrahim et al., 2020; Jikamo et al., 2021; Mbada et al., 2015; Mgbeojedo et al., 2022; Duracinsky et al., 2012; Onagbiye et al., 2018; Uwizihiwe et al., 2022; Kidayi et al., 2023; Kondo et al., 2023; El Fakir et al., 2014a; Olasehinde et al., 2024; Nkurunziza et al., 2016; Farid et al., 2023; Odetunde et al., 2020; Araya et al., 2019; El Alami et al., 2021; Osman et al., 2018; Smith and Morris-Eyton, 2023; Westmoreland et al., 2018; Guermazi et al., 2012; Ohrnberger et al., 2020; Okello et al., 2018; Owolabi, 2010; Kulich et al., 2008; Getu et al., 2022; Gadisa et al., 2019; Gqada et al., 2021), was documented in 31 (91.2%) tools. Test–retest reliability and other indicators of stability over time were reported in 26 (76.5%) tools (Ibrahim et al., 2020; Jikamo et al., 2021; Mbada et al., 2015; Mgbeojedo et al., 2022; Ravens-Sieberer et al., 2010; Duracinsky et al., 2012; Brandt et al., 2016; Borissov et al., 2022; Kondo et al., 2023; El Fakir et al., 2014a; Olasehinde et al., 2024; Nkurunziza et al., 2016; Farid et al., 2023; El Fakir et al., 2014b; Odetunde et al., 2020; Araya et al., 2019; Osman et al., 2018; Smith and Morris-Eyton, 2023; Guermazi et al., 2012; Scott et al., 2017; Owolabi, 2010; Kulich et al., 2008; Getu et al., 2022). Known-groups validity was assessed in 22 (64.7%) measurement tools (Colbourn et al., 2012; Bowden et al., 2002; Namisango et al., 2007; Reba et al., 2019; Younsi and Chakroun, 2014; Ibrahim et al., 2020; Jikamo et al., 2021; Mbada et al., 2015; Duracinsky et al., 2012; Uwizihiwe et al., 2022; Kidayi et al., 2023; Brandt et al., 2016; Borissov et al., 2022; El Fakir et al., 2014a; Nkurunziza et al., 2016; Farid et al., 2023; Araya et al., 2019; El Alami et al., 2021; Westmoreland et al., 2018; Scott et al., 2017; Okello et al., 2018; Owolabi, 2010; Kulich et al., 2008; Getu et al., 2022; Gadisa et al., 2019). Only 13 (38.2%) tools measured responsiveness (Bowden et al., 2002; Borissov et al., 2022; El Fakir et al., 2014a; Odetunde et al., 2020; Osman et al., 2018; Smith and Morris-Eyton, 2023; Owolabi, 2010; Gadisa et al., 2019). Finally, ceiling and/or floor effects were documented in 21 (61.8%) tools, with some showing minimal issues (Colbourn et al., 2012; Van Biljon et al., 2015; Ibrahim et al., 2020; Mgbeojedo et al., 2022; Muhye and Fentahun, 2023; Ravens-Sieberer et al., 2010; Uwizihiwe et al., 2022; Borissov et al., 2022; Kondo et al., 2023; El Fakir et al., 2014a; Olasehinde et al., 2024; Farid et al., 2023; El Fakir et al., 2014b; Odetunde et al., 2020; Smith and Morris-Eyton, 2023; Scott et al., 2017; Okello et al., 2018; Owolabi, 2010; Kulich et al., 2008). Other information is found in Supplementary Tables 8a–e.

## Discussion

### Summary of key findings

This systematic review identified a diverse range of generic and disease-specific HRQoL instruments developed, adapted, or validated for use across African populations between 2015 and 2025. Although several tools demonstrated acceptable psychometric performance, particularly in internal consistency and construct validity, many studies lacked comprehensive assessments of measurement properties, including responsiveness, structural validity, and test–retest reliability. Cultural and linguistic adaptation methods varied widely, and several studies reported challenges related to conceptual equivalence and contextual relevance. Overall, the evidence demonstrates growing use of PROMs in Africa but highlights persistent gaps in methodological rigor, geographical coverage, and reporting quality.

### Comparison with previous studies

More than 90% of the studies reviewed used a cross-sectional methodological design (Colbourn et al., 2012; Namisango et al., 2007; Van Biljon et al., 2015; Reba et al., 2019; Younsi and Chakroun, 2014; Jikamo et al., 2021; Mbada et al., 2015; Muhye and Fentahun, 2023; Ravens-Sieberer et al., 2010; Ehab et al., 2021; Duracinsky et al., 2012; Gqada et al., 2021; Onagbiye et al., 2018; Uwizihiwe et al., 2022; Kidayi et al., 2023; Brandt et al., 2016; Kondo et al., 2023; El Fakir et al., 2014a; Olasehinde et al., 2024; Nkurunziza et al., 2016; Farid et al., 2023; El Fakir et al., 2014b; Odetunde et al., 2020; Araya et al., 2019; El Alami et al., 2021; Osman et al., 2018; Guermazi et al., 2012), indicating a significant reliance on single-point data collection, which captures snapshots of HRQoL and limits the ability to track changes over time or assess the impact of interventions (Setia, 2016). Few studies used a longitudinal study design (Ohrnberger et al., 2020; Okello et al., 2018; Owolabi, 2010; Kulich et al., 2008; Getu et al., 2022; Gadisa et al., 2019), which is essential given the dynamic nature of the HRQoL, especially in the context of chronic diseases such as diabetes, cardiovascular diseases, and cancer. There is a need for future research to prioritize longitudinal designs to provide more robust evidence for policy and clinical decision-making. The balance between generic (Colbourn et al., 2012; Namisango et al., 2007; Van Biljon et al., 2015; Reba et al., 2019; Younsi and Chakroun, 2014; Ibrahim et al., 2020; Jikamo et al., 2021; Mbada et al., 2015; Mgbeojedo et al., 2022; Muhye and Fentahun, 2023; Ravens-Sieberer et al., 2010; Onagbiye et al., 2018; Smith and Morris-Eyton, 2023; Westmoreland et al., 2018; Guermazi et al., 2012; Scott et al., 2017; Ohrnberger et al., 2020) and disease-specific (Ehab et al., 2021; Duracinsky et al., 2012; Gqada et al., 2021; Uwizihiwe et al., 2022; Kidayi et al., 2023; Brandt et al., 2016; Borissov et al., 2022; Kondo et al., 2023; El Fakir et al., 2014a; Olasehinde et al., 2024; Nkurunziza et al., 2016; El Fakir et al., 2014b; Odetunde et al., 2020; Araya et al., 2019; El Alami et al., 2021; Osman et al., 2018; Kulich et al., 2008; Getu et al., 2022; Gadisa et al., 2019), HRQoL tools reflects a dual interest in quality-of-life assessment tools. Generic tools such as the SF-8 (Onagbiye et al., 2018), SF-12 (Younsi and Chakroun, 2014; Ibrahim et al., 2020; Ohrnberger et al., 2020), SF-36 (Mbada et al., 2015; Guermazi et al., 2012), and WHOQoL-BREF (Colbourn et al., 2012; Reba et al., 2019; Jikamo et al., 2021) offer the advantage of comparability across different populations and disease conditions. However, disease-specific tools such as the validated EORTCQLQ-BR45 (Ehab et al., 2021; Kidayi et al., 2023) for breast cancer provide more insights into their impact on the QoL of particular health conditions. The validation of these tools across diverse African populations and their translation into local languages is a significant step toward ensuring cultural and linguistic relevance (Efstathiou, 2019), this cultural adaptation of studies is essential for improving the usability and acceptability of HRQoL tools in African settings, where the health perceptions and outcomes are significantly influenced by the cultural context (Guillemin et al., 1993). For instance, adaptations of the Hausa SF-12 (Ibrahim et al., 2020) and Igbo OPQoL-35 (Mgbeojedo et al., 2022) highlight efforts to bridge the gap between culture and language, ensuring that HRQoL assessments are grounded in the lived experience of African populations. This cultural adaptation helps inform healthcare policies and improve outcomes, which could be tailored to the specific needs of the African population (Kaplan and Hays, 2022).

In this review, Southern (Van Biljon et al., 2015; Ravens-Sieberer et al., 2010; Gqada et al., 2021; Onagbiye et al., 2018; Brandt et al., 2016; Smith and Morris-Eyton, 2023; Scott et al., 2017; Kulich et al., 2008) and Eastern Africa (Namisango et al., 2007; Uwizihiwe et al., 2022; Kidayi et al., 2023; Borissov et al., 2022; Kondo et al., 2023; Araya et al., 2019; Okello et al., 2018; Getu et al., 2022; Gadisa et al., 2019) were the most represented African regions, indicating a concentration of research efforts in South and East Africa. This regional bias may reflect disparities in research capacity and funding across other African regions, such as Western Africa (Ibrahim et al., 2020; Mbada et al., 2015; Mgbeojedo et al., 2022; Duracinsky et al., 2012; Olasehinde et al., 2024; Odetunde et al., 2020; Owolabi, 2010) and Northern Africa (Younsi and Chakroun, 2014; Ehab et al., 2021; Guermazi et al., 2012), which were underrepresented. These disparities highlight the need for equitable distribution of research efforts to ensure HRQoL findings are generalizable across different African regions (Mberu et al., 2014).

There were few generic or disease-specific HRQoL measures for children and adolescents (Ravens-Sieberer et al., 2010; Borissov et al., 2022; Westmoreland et al., 2018; Scott et al., 2017), unlike in adult populations (Colbourn et al., 2012; Namisango et al., 2007; Reba et al., 2019; Younsi and Chakroun, 2014; Ibrahim et al., 2020; Jikamo et al., 2021; Mbada et al., 2015; Mgbeojedo et al., 2022; Muhye and Fentahun, 2023; Ehab et al., 2021; Duracinsky et al., 2012; Gqada et al., 2021; Onagbiye et al., 2018; Uwizihiwe et al., 2022; Kidayi et al., 2023; Brandt et al., 2016; Kondo et al., 2023; El Fakir et al., 2014a; El Fakir et al., 2014b; Odetunde et al., 2020; Araya et al., 2019; El Alami et al., 2021; Osman et al., 2018; Smith and Morris-Eyton, 2023; Guermazi et al., 2012; Scott et al., 2017; Owolabi, 2010; Kulich et al., 2008; Getu et al., 2022; Gadisa et al., 2019), indicating a gap in HRQoL research among pediatric populations. In Africa, there is a high burden of childhood diseases such as malaria, malnutrition, and other diseases among children and adolescents, which implies that there is an urgent need to validate HRQoL tools to reflect the QoL of younger populations (Akombi et al., 2017). Additionally, hospital-based validation studies (Colbourn et al., 2012; Namisango et al., 2007; Van Biljon et al., 2015; Reba et al., 2019; Ibrahim et al., 2020; Jikamo et al., 2021; Mbada et al., 2015; Ehab et al., 2021; Duracinsky et al., 2012; Gqada et al., 2021; Uwizihiwe et al., 2022; Kidayi et al., 2023; Brandt et al., 2016; Borissov et al., 2022; Kondo et al., 2023; El Fakir et al., 2014a; Olasehinde et al., 2024; Nkurunziza et al., 2016; Farid et al., 2023; El Fakir et al., 2014b; Odetunde et al., 2020; Araya et al., 2019; El Alami et al., 2021; Osman et al., 2018; Smith and Morris-Eyton, 2023; Westmoreland et al., 2018; Okello et al., 2018; Owolabi, 2010; Kulich et al., 2008; Getu et al., 2022; Gadisa et al., 2019) were predominant over community-based validation studies (Younsi and Chakroun, 2014; Mgbeojedo et al., 2022; Muhye and Fentahun, 2023; Ravens-Sieberer et al., 2010; Onagbiye et al., 2018; Guermazi et al., 2012; Scott et al., 2017; Ohrnberger et al., 2020), suggesting potential bias in recruitment because hospital-based samples may not fully represent the broader population. Community-based studies are essential for capturing the HRQoL of individuals who may not have access to formal healthcare services, particularly in rural areas. Additionally, in the psychometric evaluation, this review applied the COSMIN framework to summarize the methodological rigor of included instruments. The HRQoL tools showed strong validation practices for core psychometric properties, although responsiveness and discriminatory capacity across groups were assessed in fewer studies, highlighting areas for future methodological focus (Finch et al., 2015; Mateen et al., 2017). More than half of the studies reported sample size limitations as a major downside of their research, because small sample sizes can reduce statistical power and the ability to detect significant relationships between groups (Serdar et al., 2021). Therefore, a larger sample cohort enhances the generalisability of the findings. From a policy perspective, integration of HRQoL tools into healthcare systems is critical for advancing patient-centered care and informing resource allocation. HRQoL data can provide valuable insights into the effectiveness of interventions, the burden of disease, and the unmet needs of patients, thereby guiding the development of more responsive and equitable healthcare policies (WHO, 2020b). Generic tools, such as WHOQOL-BREF, SF-36, SF-12, and SF-8, have been validated across multiple populations and conditions, further supporting their utility in both clinical and research settings.

### Strengths and limitations of the included studies

The included studies offered valuable insights into efforts to adapt and validate HRQoL tools across multiple African regions. Strengths included the use of established theoretical frameworks for adaptation, the engagement of bilingual experts, and the inclusion of diverse clinical populations. However, limitations were common. Many studies assessed only a narrow subset of psychometric properties, cultural adaptation methods were inconsistently applied, sample sizes were sometimes insufficient for factor analysis, and several papers lacked detailed reporting required for COSMIN-based appraisal. These gaps highlight the need for more rigorous, standardized approaches to instrument validation in African contexts.

### Strengths and limitations of this review

This review followed PRISMA guidelines and used a registered PROSPERO protocol, enhancing transparency and reproducibility. Comprehensive screening, duplicate review, and structured data extraction further strengthen the credibility of the findings. However, the review has limitations. Conducting the search across PubMed, Web of Science, Scopus, and gray literature while restricting to English-language publications may have excluded relevant studies, particularly those published locally or in French, Arabic, or Portuguese. Additionally, heterogeneity in validation methodologies limited the feasibility of meta-analysis, making a narrative synthesis necessary instead. Despite these limitations, this review provides the most up-to-date and comprehensive mapping of HRQoL measurement validation in African populations.
