# Supplementary material for: Measuring health-related quality of life in Africa: a systematic review of validated disease-specific and generic measurement tools
Source: Front Psychol. 2026 Jan 7;16:1667712. doi: 10.3389/fpsyg.2025.1667712 (PMC12819306; doi:10.3389/fpsyg.2025.1667712)
Supplement: Supplementary file 1 [file Supplementary_file_1.pdf]

Measuring health-related quality of life in Africa: a systematic review of validated disease-specific and generic measurement tools

Search Strategy

The research question contains several key concepts:

1. Tools: Instruments, scales, or measures.
2. Health-Related Quality of Life (HRQoL).
3. Measurement: Quantitative or qualitative evaluation.
4. Africa: Geographical region of interest.

Search Terms

For each key term, synonyms and related terms to be used are:

Key Term	Synonyms and Related Terms
Tools	Instrument, scale, measure, questionnaire, assessment tool, survey
HRQoL	Health-related quality of life, HRQoL, QoL, quality of life, health preference
Measurement	Assessment, evaluation, measurement, validation, development
Africa	Africa, Sub-Saharan Africa, African countries, African region

Search Strings

Search strings was generated from combining terms using Boolean operators (AND, OR) and adopt truncation (*) and wildcards (?) where appropriate as:

("tool" OR "instrument*" OR "scale*" OR "questionnaire*" OR "measure*" OR "assessment tool*" OR "survey*")* ***AND***
("health-related quality of life" OR "HRQoL" OR "QoL" OR "quality of life" OR "health preference)* ***AND***
("measurement" OR "assessment" OR "evaluation" OR "validation" OR "development") ***AND***
("Africa" OR "Sub-Saharan Africa" OR "African countries" OR "African region")

Database-Specific Search Strings

1. PubMed *(supports MeSH terms and free-text searches)*

(("tool" OR "instrument*" OR "scale*" OR "questionnaire*" OR "measure*" OR "assessment tool*" OR "survey*"[Title/Abstract])* ***AND*** *("health-related quality of life"[MeSH Terms] OR "HRQoL"[Title/Abstract] OR "quality of life"[Title/Abstract] OR "health preference*"[Title/Abstract])* ***AND*** *("measurement"[Title/Abstract] OR "assessment"[Title/Abstract] OR "evaluation"[Title/Abstract] OR "validation"[Title/Abstract] OR "development"[Title/Abstract])* ***AND*** *("Africa"[MeSH Terms] OR "Africa"[Title/Abstract] OR "Sub-Saharan Africa"[Title/Abstract] OR "African countries"[Title/Abstract])) OR "African region"[Title/Abstract])) NOT (("QALY"[MeSH Terms] OR "EQ-5D*"[Title/Abstract]))*

2. Web of Science *(allows Boolean searches across titles, abstracts, and keywords)*

TS=("tool" OR "instrument*" OR "scale*" OR "questionnaire*" OR "measure*" OR "assessment tool*" OR "survey*")* ***AND***
TS=("health-related quality of life" OR "HRQoL" OR "QoL" OR "quality of life" OR "health preference)* ***AND***
TS=("measurement" OR "assessment" OR "evaluation" OR "validation" OR "development") ***AND***
TS=("Africa" OR "Sub-Saharan Africa" OR "African countries" OR "African region")

TS: Topics

3. SCOPUS *(enables comprehensive search using proximity operators)*

(TITLE-ABS-KEY("tool" OR "instrument*" OR "scale*" OR "questionnaire*" OR "measure*" OR "assessment tool*" OR "survey*")* ***AND***
TITLE-ABS-KEY("health-related quality of life" OR "HRQoL" OR "QoL" OR "quality of life" OR "health preference)* ***AND***
TITLE-ABS-KEY("measurement" OR "assessment" OR "evaluation" OR "validation" OR "development") ***AND***
TITLE-ABS-KEY("Africa" OR "Sub-Saharan Africa" OR "African countries" OR "African region"))

TITLE-ABS-KEY: Title-Abstract-Keywords
