# Supplementary material for: Measuring health-related quality of life in Africa: a systematic review of validated disease-specific and generic measurement tools
Source: Front Psychol. 2026 Jan 7;16:1667712. doi: 10.3389/fpsyg.2025.1667712 (PMC12819306; doi:10.3389/fpsyg.2025.1667712)
Supplement: Supplementary file 2 [file Data_Sheet_1.PDF]

Table 8a: Summary of HRQoL tools following the COSMIN guideline

S/No	Tools (Authors)	Feasibility	Reliability	Internal Consistency	Content Validity	Construct Validity	Known-Groups Validity	Responsiveness	Ceiling Effects
1.	WHOQOL-OLD(33,40)	No major issue reported, 100% response rate(33,40)	For subscales: a=0.759-0.902, total score=0.882[19]; For subscales: a= 0.902-0.932, total score= 0.963(40)	Good[19]; excellent[31]	Not explicitly mentioned; Minor adjustment for cultural relevance	(RMSEA = 0.050, CFI = 0.97) (82); (RMSEA = 0.047, CFI = 0.975) (40).	Not assessed(33,40)	Not assessed(33,40)	Not explicitly mentioned(82); Minimal (1.8–7.7% for ceiling effects; 0–2.9% for floor effects)(40).
2.	STI-HRQoL longform 37 items(60)	Minor adjustment made based on pilot feedback	Excellent test-retest reliability (ICC = 0.94)	High a= 0.94 (for total score) and 0.92-0.94 (For subscales)	Items were developed via expert panel and pilot testing	PCA confirmed 3 dimensions.	Not explicitly assessed	Clinically Significant change threshold (Coefficient of repeatability= ± 12.04%	Minimal outliers
3.	STI-HRQoL-Shortform 25 items(60)	Quick screening	Excellent test-retest reliability (ICC = 0.94)	High a= 0.94 (for total score) and 0.92-0.94 (For subscales)	Retained items with highest inter-item correlations	PCA confirmed 3 dimensions	Not explicitly assessed	Clinically Significant change threshold (Coefficient of repeatability= ± 12.04%	Minimal outliers
4.	WHOQOL-BREF(13,34,37)	High response rate(13,34,37)	Not assessed (13,34); Test-retest ICC=0.78(37)	Subscales; a= 0.58-0.84(34); a=0.80-0.91(37);0.69-0.83(13)	Not reported(34); Item level (0.86-1.00)(37); Expert panel reviewed translations(13)	CFA showed acceptability (CFI = 0.94, RMSEA = 0.055)(34); CFA showed poor fit (CFI = 0.87, RMSEA = 0.23)(37); Not performed(13)	Physical and psychological domains significantly contributed to overall QOL(34); Depression and disability weights discriminated domain scores(13,37)	Not assessed(13,34,37)	Not explicitly reported(34,37); Ceiling effects noted in psychological and social domain(13).
5.	PROMIS-25(61)	Acceptable	Test-retest not assessed	a= 0.71-0.90 for the sub scales	Expert panel reviewed translations	Structural validity supported by inter-item correlations and PCA	Discriminated between anaemic/ non-anaemic and performance status groups	Not assessed	High in the pain domains

Table 8b: Summary of HRQoL tools following the COSMIN guideline

S/No	Tools (Authors)	Feasibility	Reliability	Internal Consistency	Content Validity	Construct Validity	Known-Groups Validity	Responsiveness	Ceiling Effects
6.	SF-12(35,36,64)	High acceptability	a=0.72- 0.73 but Test-retest reliability not assessed(35); ICC: 0.79 (PCS-12), 0.85 (MCS-12)(36); α=0.9 and, 0.72 (second factor)(64)..	Satisfactory for all domains(35,36). Satisfactory for the mental health domain but weaker for physical health(64)	Item correlation between mental and physical health domain(35,64); two-factor structure retained after modifications)(36)	Strong correlations between items (PCS-12/MCS-12)(35); Convergent validity: 91% success rate. Divergent validity: 83% success rate(36); Better construct validity for Malawi-weighted mental health (rs = 0.675 vs. 0.495)(64)	Tunisian and Hausa versions discriminated well by demographic factors(35,36); Malawian study focused on factor structure(64)	Not assessed	Not observed(35) (Younsi); Floor and ceiling effects observed(36); Not reported(64)
7.	SF-36(38,62,65)	Good(38,62); Adaptable(65)	ICC ranging from 0.636 to 0.843 for scales and 0.783 to 0.851 for domains(38). ICC= an excellent score of 0.98(62) Acceptable reliability, with intraclass correlation coefficients (ICC)(65)	Specific values were not reported(38) a= 0.94 overall, for subscales=from 0.72 to 0.89(62); a= 0.79 for overall scales, the subscales a= 0.71-0.81(65)	Items were conceptually equivalent and culturally appropriate(38); No specific details, but the adaptation process and pilot study suggest that the content was relevant and appropriate for the Tunisian population(62); not explicitly discussed(65)	The results showed satisfactory convergent validity ranging from 0.421 to 0.907(38); It Convergent validity showed strong correlations and divergent validity showed fair to poor correlations with the sociodemographic(62); CFA was used to test the unidimensionality(65)	Tested by comparing scale and domain scores across different gender and age groups(38); No specific details(62); Differences in scores based on clinical outcomes(65)	No specific information(38); No specific information(62); No specific information(65)	No specific information(38); Not mentioned(62); The SF-36 exhibited ceiling effects in several subscales, including bodily pain, mental health, social functioning, and physical health(65)
8.	MVQOLI(17)	Good	Good reliability, with a= 0.85	The internal consistency of the MVQOLI-M was strong overall, but the physical symptom domain initially had weak internal consistency	All items and domains were rated content valid (CVR > 0.62	Convergent validity with a strong correlation score of (r=0.580,P<0.0005*r*=0.580,*P*<0.0005). Divergent validity was shown by low correlation with Karnofsky Performance Status scores (r=0.25,P<0.0005*r*=0.25,*P*<0.0005).	Religious affiliation significantly predicted QOL scores	Not explicitly tested in this study	Not explicitly mentioned in this study
9.	I-OPQOL(39)	Feasible not difficult to administer	Good (a=0.78)	Good indicating homogeneity	Minor modification to three items for cultural relevance	Excellent, PCA supported a 9-factor structure explaining 72.10% of the variance.	Not explicitly tested in this study	Not explicitly tested in this study	No ceiling or floor effects were observed
10.	EQ-5D-Y(41,63)	Good	Adequate (κ = 0.365–0.653(63); (κ = 0.22–0.67)(41)	Not Reported	Limited evidence(63); Not assessed(41)	Sufficient	Yes	Sufficient ((0.36–0.43)(63); Not assessed(41)	High

Table 8c: Summary of HRQoL tools following the COSMIN guideline

S/No	Tools (Authors)	Feasibility	Reliability	Internal Consistency	Content Validity	Construct Validity	Known-Groups Validity	Responsiveness	Ceiling Effects
11.	EORTC QLQ-BR45(42,47,68)	High	Test-retest (a=0.77) for all domains(68); Not reported(42,47)	a= 0.80 (all domains), a=0.66 (arm symptoms)(68); a=0.7 for most scales except a=0.594 for the systemic therapy side effects scale(47); a= >0.7 for most scales except body image (a=0.51) and systemic therapy (a=0.63)(42)	I-CVI: 0.83–1; S-CVI/Ave: 0.94; CVR: 0.76(68); Not explicitly reported(42,47)	For Convergent validity: Most scales met the criteria(42,68); For divergent validity: 88.7%-100%(42,68); Strong correlations between QLQ-C30 and BR45 domains; CFA showed good model fit(47)	Significant difference by the sociodemographic factors, performance status, cancer stage(47,68); Not explicitly assessed(42)	Not assessed	Not reported
12.	PROQOL-HIV(43)	High	ICC=0.86	Subscales: 0.77-0.89	High (Item reduction based on relevance)	High	Differences by CD4 count symptoms	Not assessed	Minimal (Items with > 75% saturation removed)
13.	EORTC QLQPAN26(44)	High	Not reported	0.88 (isiXhosa), 0.89 (Afrikaans)	High	Moderate (lower Cronbach alpha for subscales)	Not assessed	Not assessed	Low (100% completion for most items)
14.	SF-8(45)	High	Fair to moderate reliability	0.87 (Setswana), 0.89 (English)	High (Cultural adaptation)	High	Not assessed	Not assessed	Not reported
15.	KCCQ(65)	High	Poor (ICC: 0.08-0.45 across subscales	Excellent: a= 0.87 for overall scores; 0.79-0.86 for subscales score	High	Correlation with clinical outcomes.	Worse KCCQ scores predicted mortality	Not explicitly assessed	Minimal (≤ 3% for most subscales)
16.	D39(46)	Translated and culturally adapted	Not explicitly tested	a= 0.72-0.90 for subscales and 0.7 for overall scales	Expert panel ensured semantic and conceptual equivalence	CFA showed satisfactory fit (CFI=0.93, RMSEA=0.05)	Detected differences by gender, education and health status	Not explicitly tested	No significant ceiling or floor effect reported
17.	HRQOLISP(66)	Reduced from 102 to 40 items for easier clinical use	Good test-retest reliability (weighted k> 0.75)	a= 0.76-0.89 (physical sphere), 0.78 (spiritual sphere)	Strong correlation with originall HRQOLISP	Convergent validity within spheres and discriminant validity across spheres	Discriminated between stroke and healthy groups	Predicted good sensitivity to change	Minimal (<2015 in most domains)

Table 8d: Summary of HRQoL tools following the COSMIN guideline

S/No	Tools (Authors)	Feasibility	Reliability	Internal Consistency	Content Validity	Construct Validity	Known-Groups Validity	Responsiveness	Ceiling Effects
18.	EORTC-QLQ-C30(47,57,58,69)	High compliance(57,58,69); No major issues reported(47)	ICC (0.78-0.92)(57); Not assessed(58,69); Not explicitly reported(47)	a= ≥ 0.70 for all scales except physical functioning(57); a= ≥ 0.70 for all scales(58); a=≥ 0.70 for most scales except cognitive function and body image(47)	Forward-backward translation(57,58,69); Adhered to EORTC translation(47)	Convergent/Discriminant validity confirmed(57,58,69); Significant correlation between EORTC-QLQ-C30 and EORTC-QLQ-BR45 dimensions(47)	Differentiated stoma vs. non-stoma, colon vs rectal cancer (p<0.05)(57); Differentiated FIGO stages, ECOG-PS, and treatment groups (p<0.05)(58); Differentiated chemotherapy-induced changes (p<0.05); Not explicitly tested(47)	Not assessed(57,58); significant score changes(69); Not assessed(47)	Not reported(57,58,69[illegible] Not reported(47)[illegible]
19.	BREAST-Q questionnaire(52)	No major issues reported (49)	ICC: Moderate (0.41-0.64)	Strong domain (Sexual wellbeing); a= 0.98-0.99; Moderate domain (Satisfaction with breast): a=0.43-0.63	Forward/back translation and cultural adaptation (pain descriptors was adjusted)	Strong correlations between domains	Not explicitly tested	Not assessed	Observed in satisfaction domain
20.	SF-NDI(53)	No major issues reported	Test-retest reliability (Spearman's correlation): 0.89.	First time α= 0.96, second time: α= 0.95.	Forward/backward translation, pilot testing and cultural adaptation	The AUC of 0.89 with no statistical difference from SF-LDQ, p=0.35	Significant differences in SF-NDI scores between mild/severe dyspepsia	Not assessed	Not reported
21.	Modified pTESS(54)	Appropriate for for children/adolescnts	Excellent test-retest reliability (ICC>0.8)	High a= >0.9	Culturally adapted	EFA for pTESS-leg as other versions involved insufficient numbers of participants. extracted 3 factors	Lower scores for tibial tumors versus femur at p=0.002)	Not assessed	None observed
22.	Modified TESS(54)	Appropriate for adults	Excellent test-retest reliability (ICC>0.8)	High a= >0.9	Culturally adapted (e.g sexual activities rephrased conservatively	Insufficient sample size for EFA: relied on inter-item correlations (Acceptable 0.3-0.7)	Scores improved in respondents that have had surgery greater than 1 year at (p<0.001)	Not assessed	None observed
23.	EQ-5D-3L(43,55)	High	Test-retest reliability ICC>0.70 (Moderate to high)(55); Not explicitly mentioned(43)	**low to moderate** (Cronbach's α ~0.5–0.7)(51); Not applicable(43)	High	Strong correlation with other HRQoL measures(51); Moderate correlation with PROQOL-HIV global score(43)	It distinguished between healthy vs chronically ill groups(51); Not explicitly tested(43)	For acute conditions: moderate to high; for chronic diseases: Lower(51)(El Fakir); Not evaluated(43)	High(51); Not explicitly mentioned(43)
24.	Skindex-16(55)	Easy to administer	High test-retest reliability (ICC > 0.80 in most studies).	a= >0.80 for all domains	It Covers key domains (symptoms, psychosocial impact, functioning)	Correlates well with the generic QoL measures used in the study	Distinguishes between patients with mild vs. severe skin conditions (e.g., psoriasis, eczema).	Sensitive to clinical changes	Minimal ceiling effects

Table 8e: Summary of HRQoL tools following the COSMIN guideline

S/No	Tools (Authors)	Feasibility	Reliability	Internal Consistency	Content Validity	Construct Validity	Known-Groups Validity	Responsiveness	Ceiling Effects
25.	SS-QoL 2.0(56)	Successfully adapted to the Igbo language	Excellent test-retest reliability (ICC = 0.91 overall; domain ICCs = 0.50–0.84).	Cronbach's alpha = 0.69–0.87 for overall except vision domain a= 0.21	Expert committee confirmed semantic, idiomatic, and conceptual equivalence with the original English version.	Moderate to high correlations (r = 0.58–0.87) between Igbo and English versions for all domains.	No significant differences by gender or age groups (p > 0.05).	Detected improvements post-treatment (effect sizes: 0.60–1.49).	<20% for the floor effects while >20% for . Ceiling effects for the domains
26.	EORTC QLQ-CX24(57)	High compliance with no missing responses	a= 0.70-0.96	All scales met the threshold (α ≥ 0.70); body image scale showed very high consistency (α = 0.96).	Confirmed by pilot testing and expert review culturally adapted for Ethiopian patients	Convergent (r ≥ 0.40) and discriminant validity confirmed via multitrait scaling analysis.	Discriminated between FIGO stages and ECOG-PS	Not assessed in this study	Not explicitly reported
27.	Quality of Life in Reflux and Dyspepsia (QOLRAD)(67)	High compliance	Test-retest reliability (ICC): 0.41–0.82	a= 0.79–0.95	Confirmed through linguistic validation and pilot testing.	Convergent validity: Strong correlations with SF-36 domains	Discriminated between symptom severity groups.	Not explicitly assessed in this study	Not reported
28.	Prolapse quality-of-life questionnaire (P-QOL)(48)	High compliance	Test-retest reliability: Median agreement of 69.2%	High a= 0.94 but for severity measures α=0.36–0.46	Confirmed through expert review and pilot testing;	Symptomatic participants scored higher (poorer QOL) than asymptomatic (p<0.05)	Discriminated between pelvic organ prolapse (POP)stages	Not assessed	Not reported
29.	PedsQL™ FIM (acute version)(49,54)	Feasible but some participants required clarification(54); Good (49)	PedsQL™ was used as a reference for validation for pTESS/TESS(54) (Farid); Test-retest reliability was moderate to excellent (ICC = 0.63–0.91) for each domain but excellent reliability (ICC = 0.91) for the overall(49)	PedsQL™ was used as a benchmark for comparison with pTESS/TESS(54); α = 0.94–0.97) for total score but most subscales (α = 0.80–0.96), except for the Communication subscale (α = 0.59–0.70)(49)	No direct content validity analysis was performed for PedsQL™ in this study(54); The tool was culturally adapted and translated into Amharic, with modifications to improve clarity(49)	Moderate to strong correlations (r = 0.55–0.86) were found between PedsQL™ and pTESS/TESS scores, supporting convergent validity. Factor analysis was not performed for PedsQL™ in this study(54); CFA supported the 8-factor structure (RMSEA = 0.068, CFI = 0.975, TLI = 0.973, SRMR = 0.067)(49).	PedsQL™ scores were lower in patients with bone sarcomas compared to healthy children, aligning with expectations(54); Significantly lower functioning (higher impact) than those of children with physical health conditions(49)	Not assessed	Not observed

Table 8f: Summary of HRQoL tools following the COSMIN guideline

S/No	Tools (Authors)	Feasibility	Reliability	Internal Consistency	Content Validity	Construct Validity	Known-Groups Validity	Responsiveness	Ceiling Effects
30.	WHOQOL-HIV BREF (50)	High	Excellent test-retest reliability (ICC: 0.91–0.92	High a= 0.89-0.90	Ensured through meticulous translation and back-translation processes following WHO guidelines.	Convergent and discriminant validity were satisfactory.	Not explicitly assessed	Not evaluated	Observed in domains like Physical pain, HIV symptoms, Stigma and Death worries while floor effects was noted in sexual activity
31.	EORTC QLQ-BR-23(51,69)	Interviewer administered due to low literacy rates	ICC for test-retest ranged from 0.68 (sexual enjoyment) to 0.85 (body image)(51); Not assessed(69)	a= 0.50-0.76 for subscales(51); a= 0.51-0.96 for subscales(69)	Adapted culturally	100% item-convergence for body image and arm symptoms, but only 50% for breast symptoms(51); Strong correlations between systemic therapy side effects and EORTC QLQ-C30 scales(69)	Not explicitly tested	Not assessed(51); Significant changes in scores after chemotherapy(69)	High ceiling effect for body image (39%), future perspective (24.8%), and breast symptoms (25.7%)(51); Not reported(69)
32.	GOHAI(59)	Good	Excellent test-retest reliability (ICC = 0.893).	A=0.711 (with denture experience), while a=0.713 (without denture experience)	Culturally adapted with modifications for Sudanese dialect	Significant correlations with self-rated oral health	Could not distinguish between patients with/without denture experience	Detected significant improvements post-treatment	Not explicitly reported.
33.	OHIP-14(59)	Good	Not explicitly reported	a= 0.638–0.718 for subscales	Minor adjustments for local context.	Significant correlations with denture satisfaction (DS) and self-rated general health	Could not distinguish between patients with/without denture experience	Detected significant improvements post-treatment	Not explicitly reported.
34.	KENQOL(14)	Good	Addressed wording clarity, jargon, and double negatives to minimize response variability	Not explicitly measured quantitatively but qualitatively ensured questions were unambiguous	Expert reviews and respondent feedback	Convergent validity: Correlated responses with self-rated health and local health concepts. Discriminant validity: Confirmed questions distinguished between groups	Compared responses across subgroups (age, gender, socioeconomic status)	Assessed sensitivity to change via hypothetical scenarios	Not explicitly reported
