# Supplementary material for: Measuring health-related quality of life in Africa: a systematic review of validated disease-specific and generic measurement tools
Source: Front Psychol. 2026 Jan 7;16:1667712. doi: 10.3389/fpsyg.2025.1667712 (PMC12819306; doi:10.3389/fpsyg.2025.1667712)
Supplement: Supplementary file 3 [file Data_Sheet_2.PDF]

Measuring health-related quality of life in Africa: a systematic review of validated disease-specific and generic measurement tools

Table 2a: Quality Assessment of the Included Articles

S/No	First Author	Were the criteria for inclusion in the sample clearly defined?	Were the study subjects and the setting described in detail?	Was the exposure measured in a valid and reliable way?	Were objective, standard criteria used for measurement of the condition?	Were confounding factors identified?	Were strategies to deal with confounding factors stated?	Were the outcomes measured in a valid and reliable way?	Was appropriate statistical analysis used?
1	Van Biljon, et al(1)	Yes	Yes	Yes	Yes	Yes	Yes	Yes	Yes
2	Smith L et al(2)	Yes	Yes	Yes	Yes	Yes	Yes	Yes	Yes
3	Reba et al(3)	Yes	Yes	Yes	Yes	Yes	Yes	Yes	Yes
4	Westmoreland et al(4)	No	No	Unclear	No	Unclear	Unclear	Unclear	Unclear
5	Younsi & Chakroun (5)	Yes	No	Yes	Yes	Unclear	Unclear	Yes	Yes
6	Ibrahim et al(6)	Yes	Yes	Yes	Yes	Yes	Yes	Yes	Yes
7	Jikamo et al(7)	Yes	Yes	Yes	Yes	Unclear	Unclear	Yes	Yes
8	Mbada et al(8)	Yes	Yes	Yes	Yes	Yes	Yes	Yes	Yes
9	Namisango et al(9)	Yes	Yes	Yes	Yes	No	No	Yes	Yes
10	Colbourn et al(10)	Yes	Yes	Yes	Yes	No	No	Yes	Yes
11	Guermazi et al(11)	No	No	Yes	Yes	No	No	Yes	Yes
12	Mgbeojedo et al(12)	Yes	Yes	Yes	Yes	Not applicable	Not applicable	Yes	Yes
13	Muhye (13)	Yes	Yes	Yes	Yes	Not applicable	Not applicable	Yes	Yes

S/No	First Author	Were the criteria for inclusion in the sample clearly defined?	Were the study subjects and the setting described in detail?	Was the exposure measured in a valid and reliable way?	Were objective, standard criteria used for measurement of the condition?	Were confounding factors identified?	Were strategies to deal with confounding factors stated?	Were the outcomes measured in a valid and reliable way?	Was appropriate statistical analysis used?
14	Scott et al(14)	Yes	Yes	Yes	Yes	No	No	Yes	Yes
15	Ravens-Sieberer et al(15)	Yes	Yes	Yes	Yes	No	No	Yes	Yes
16	Ehab et al(16)	Yes	Yes	Yes	Yes	Not applicable	Not applicable	Yes	Yes
17	Duracinsky et al(17)	No	Yes	Yes	Yes	Unclear	Unclear	Yes	Yes
18	Gqada et al(18)	No	No	Yes	Yes	No	No	Yes	Yes
19	Onagbiye et al(19)	Yes	Yes	Yes	Yes	Not applicable	Not applicable	Yes	Yes
20	Ohrnberger et al.(20)	Yes	Yes	Yes	Yes	No	No	Yes	Yes
21	Okello et al(21).	Yes	Yes	Yes	Yes	Unclear	Unclear	Yes	Yes
22	Uwizihiwe et al(22).	Yes	Yes	Yes	Yes	Not applicable	Not applicable	Yes	Yes
23	Owolabi et al(23)	Yes	Yes	Yes	Yes	No	No	Yes	Yes
24	Kidayi et al(24)	Yes	Yes	Yes	Yes	No	No	Yes	Yes
25	Brandt et al(25)	Yes	No	Yes	Yes	No	No	Yes	Yes
26	Kulich et al(26)	Yes	Yes	Yes	Yes	No	No	Yes	Yes
27	Borissov A et al(27)	Yes	Yes	Yes	Yes	Yes	Yes	Yes	Yes
28	Kondo et al(28)	Yes	Yes	Yes	Yes	No	No	Yes	Yes

S/No	First Author	Were the criteria for inclusion in the sample clearly defined?	Were the study subjects and the setting described in detail?	Was the exposure measured in a valid and reliable way?	Were objective, standard criteria used for measurement of the condition?	Were confounding factors identified?	Were strategies to deal with confounding factors stated?	Were the outcomes measured in a valid and reliable way?	Was appropriate statistical analysis used?
29	El Fakir et al(29)	No	No	Yes	Yes	No	No	Yes	Yes
30	Getu et al(30)	Yes	Yes	Yes	Yes	Yes	Yes	Yes	Yes
31	Olasehinde et al(31)	Yes	Yes	Yes	Yes	Not applicable	Not applicable	Yes	Yes
32	Nkurunziza et al(32)	Yes	Yes	Yes	Yes	Not applicable	Not applicable	Yes	Yes
33	Farid et al(33)	Yes	No	Yes	Yes	No	No	Yes	Yes
34	El Fakir et al(34)	Yes	Yes	Yes	Yes	Not applicable	Not applicable	Yes	Yes
35	Odetunde et al(35)	Yes	Yes	Yes	Yes	Not applicable	Not applicable	Yes	Yes
36	Araya et al(36)	Yes	Yes	Yes	Yes	No	No	Yes	Yes
37	El Alami et al(37)	Yes	Yes	Yes	Yes	Unclear	Unclear	Yes	Yes
38	Gadisa et al(38)	Yes	Yes	Yes	Yes	Not applicable	Not applicable	Not applicable	Yes
39	Osman et al(39)	Yes	Yes	Yes	Yes	Yes	Yes	Yes	Yes
40	Bowden et al(40)	No	No	Yes	Yes	Not applicable	Not applicable	Yes	Not applicable

References

1. Van Biljon L, Nel P, Roos V. A partial validation of the WHOQOL-OLD in a sample of older people in South Africa. Glob Health Action. 2015 Dec;8(1):28209.

2. Smith L, Morris-Eyton H, Department of Sport and Movement Studies, Faculty of Health Sciences, University of Johannesburg, South Africa. Development, validation and reliability of the Smith Toolkit for Integrated Health Related Quality of Life (STI-HRQoL). Health Sports Rehabil Med. 2023 Mar 21;24(1):4–10.

3. Reba K, Birhane BW, Gutema H. Validity and Reliability of the Amharic Version of the World Health Organization's Quality of Life Questionnaire (WHOQOL-BREF) in Patients with Diagnosed Type 2 Diabetes in Felege Hiwot Referral Hospital, Ethiopia. J Diabetes Res. 2019 May 6;2019:1–6.

4. Westmoreland K, Reeve BB, Amuquandoh A, Van Der Gronde T, Manthalu O, Correia H, et al. Translation, psychometric validation, and baseline results of the Patient-Reported Outcomes Measurement Information System (PROMIS) pediatric measures to assess health-related quality of life of patients with pediatric lymphoma in Malawi. Pediatr Blood Cancer. 2018 Nov;65(11):e27353.

5. Younsi M, Chakroun M. Measuring health-related quality of life: psychometric evaluation of the Tunisian version of the SF-12 health survey. Qual Life Res. 2014 Sep;23(7):2047–54.

6. Ibrahim AA, Akindele MO, Ganiyu SO, Kaka B, Abdullahi BB, Sulaiman SK, et al. The Hausa 12-item short-form health survey (SF-12): Translation, cross-cultural adaptation and validation in mixed urban and rural Nigerian populations with chronic low back pain. Montazeri A, editor. PLOS ONE. 2020 May 7;15(5):e0232223.

7. Jikamo B, Adefris M, Azale T, Alemu K. Cultural adaptation and validation of the Sidamic version of the World Health Organization Quality-of-Life-Bref Scale measuring the quality of life of women with severe preeclampsia in southern Ethiopia, 2020. Health Qual Life Outcomes. 2021 Dec;19(1):239.

8. Mbada CE, Adeogun GA, Ogunlana MO, Adedoyin RA, Akinsulore A, Awotidebe TO, et al. Translation, cross-cultural adaptation and psychometric evaluation of yoruba version of the short-form 36 health survey. Health Qual Life Outcomes. 2015 Dec;13(1):141.

9. Namisango E, Katabira E, Karamagi C, Baguma P. Validation of the Missoula-Vitas Quality-of-Life Index Among Patients with Advanced AIDS in Urban Kampala, Uganda. J Pain Symptom Manage. 2007 Feb;33(2):189–202.

10. Colbourn T, Masache G, Skordis-Worrall J. Development, reliability and validity of the Chichewa WHOQOL-BREF in adults in lilongwe, Malawi. BMC Res Notes. 2012 Dec;5(1):346.

11. Guermazi M, Allouch C, Yahia M, Huissa TBA, Ghorbel S, Damak J, et al. Translation in Arabic, adaptation and validation of the SF-36 Health Survey for use in Tunisia. Ann Phys Rehabil Med. 2012 Sep;55(6):388–403.

12. Mgbeojedo UG, Ekigbo CC, Okoye EC, Ekechukwu EN, Justina Okemuo A, Ikele CN, et al. IGBO Version of the Older People's Quality Of Life Questionnaire (OPQOL-35) Is Valid and Reliable: Cross-Cultural Adaptation and Validation. Inq J Health Care Organ Provis Financ. 2022 Jan;59:00469580221126290.

13. Muhye A, Fentahun N. Validation of Quality-of-Life assessment tool for Ethiopian old age people. F1000Research. 2023 Mar 14;12:282.

14. Scott D, Ferguson GD, Jelsma J. The use of the EQ-5D-Y health related quality of life outcome measure in children in the Western Cape, South Africa: psychometric properties, feasibility and usefulness - a longitudinal, analytical study. Health Qual Life Outcomes. 2017 Jan;15(1):12.

15. Ravens-Sieberer U, Wille N, Badia X, Bonsel G, Burström K, Cavrini G, et al. Feasibility, reliability, and validity of the EQ-5D-Y: results from a multinational study. Qual Life Res. 2010 Aug;19(6):887–97.

16. Ehab BH, Hussein RRS, Abdullah EA, Ahmed ZM, Elsherbiny RM. Cultural Adaptation and Validation of the EORTC QLQ-BR45 to Assess Health-Related Quality of Life of Breast Cancer Patients. Eur Pharm J. 2021 Dec 1;68(2):41–8.

17. Duracinsky M, Lalanne C, Le Coeur S, Herrmann S, Berzins B, Armstrong AR, et al. Psychometric Validation of the PROQOL-HIV Questionnaire, a New Health-Related Quality of Life Instrument–Specific to HIV Disease. JAIDS J Acquir Immune Defic Syndr. 2012 Apr 15;59(5):506–15.

18. Gqada K, Kotze U, Soldati V, Kloppers C, Krige J, Jonas E. Translation and linguistic validation of the EORTC QLQ-PAN26 questionnaire for assessment of health-related quality of life in patients with pancreatic cancer and chronic pancreatitis into isiXhosa and Afrikaans. S Afr J Surg [Internet]. 2021 [cited 2025 Jan 24];59(4). Available from: http://ref.scielo.org/vhr3ws

19. Onagbiye SO, Moss SJ, Cameron M. Validity and reliability of the Setswana translation of the Short Form-8 health-related quality of life health survey in adults. Health SA Gesondheid [Internet]. 2018 Nov 22 [cited 2025 Jan 24];23. Available from: http://www.hsag.co.za/index.php/hsag/article/view/1092

20. Ohrnberger J, Anselmi L, Fichera E, Sutton M. Validation of the SF12 mental and physical health measure for the population from a low-income country in sub-Saharan Africa. Health Qual Life Outcomes. 2020 Dec;18(1):78.

21. Okello S, Abeya FC, Lumori BAE, Akello SJ, Moore CC, Annex BH, et al. Validation of heart failure quality of life tool and usage to predict all-cause mortality in acute heart failure in Uganda: the Mbarara heart failure registry (MAHFER). BMC Cardiovasc Disord. 2018 Dec;18(1):232.

22. Uwizihiwe JP, Lygidakis C, Bia M, Dukundane D, Asiimwe-Kateera B, Nsanzimana S, et al. Cultural adaptation and psychometric evaluation of the Kinyarwanda version of the diabetes-39 (D-39) questionnaire. Health Qual Life Outcomes. 2022 Aug 16;20(1):122.

23. Ojo Owolabi M. Psychometric Properties of the HRQOLISP-40: A Novel, Shortened Multiculturally Valid Holistic Stroke Measure. Neurorehabil Neural Repair. 2010 Nov;24(9):814–25.

24. Kidayi PL, Pakpour AH, Saboonchi F, Bray F, Manhica H, Mtuya CC, et al. Cross-Cultural Adaptation and Psychometric Properties of the Swahili Version of the European Organization for Research and Treatment of Cancer (EORTC) QLQ-BR45 among Breast Cancer Patients in Tanzania. Healthcare. 2023 Sep 5;11(18):2467.

25. Brandt C, Van Rooyen C, Cronje H. Validation of the prolapse quality of life questionnaire (P-QOL): an Afrikaans version in a South African population. South Afr J Obstet Gynaecol. 2016 Dec 12;22(2):38.

26. Kulich KR, Madisch A, Pacini F, Piqué JM, Regula J, Van Rensburg CJ, et al. Reliability and validity of the Gastrointestinal Symptom Rating Scale (GSRS) and Quality of Life in Reflux and Dyspepsia (QOLRAD) questionnaire in dyspepsia: A six-country study. Health Qual Life Outcomes. 2008;6(1):12.

27. Borissov A, Bakolis I, Tekola B, Kinfe M, Ceccarelli C, Girma F, et al. Adaptation and validation of two autism-related measures of skills and quality of life in Ethiopia. Autism. 2022 Aug;26(6):1409–22.

28. Kondo NA, Mwansisya T, Aghan E, Rwegerera G, Ratansi R. Validation of Kiswahili Version of WHOQOL-HIV BREF questionnaire among people living with HIV/AIDS in Tanzania- a cross sectional study. Pan Afr Med J [Internet]. 2023 [cited 2025 Jan 20];44. Available from: https://www.panafrican-med-journal.com/content/article/44/95/full

29. El Fakir S, Abda N, Bendahhou K, Zidouh A, Bennani M, Errihani H, et al. The european organization for research and treatment of cancer quality of life questionnaire-BR 23 breast cancer-specific quality of life questionnaire: psychometric properties in a Moroccan sample of breast cancer patients. BMC Res Notes. 2014 Dec;7(1):53.

30. Getu MA, Wang P, Kantelhardt EJ, Seife E, Chen C, Addissie A. Translation and validation of the EORTC QLQ-BR45 among Ethiopian breast cancer patients. Sci Rep. 2022 Jul 29;12(1):605.

31. Olasehinde O, Lynch KA, Goldman DA, Agodirin O, Okereke C, Wuraola FO, et al. Translation and psychometric assessment of the mastectomy module of the BREAST-Q questionnaire for use in Nigeria. J Patient-Rep Outcomes. 2024 Feb 9;8(1):17.

32. Nkurunziza A, Dusabejambo V, Everhart K, Bensen S, Walker T. Validation of the Kinyarwanda-version Short-Form Leeds Dyspepsia Questionnaire and Short-Form Nepean Dyspepsia Index to assess dyspepsia prevalence and quality-of-life impact in Rwanda. BMJ Open. 2016 Jun;6(6):e011018.

33. Farid N, Chun S, Hassanain O, Salama M, Esam E, Adel F, et al. Cross-cultural adaptation and validation of a self-reporting tool to assess health-related quality of life for Egyptians with extremity bone sarcomas in childhood or adolescence. Health Qual Life Outcomes. 2023 Jul 29;21(1):81.

34. El Fakir S, Baybay H, Bendahhou K, Obtel M, Benchat L, Mernissi FZ, et al. Validation of the Skindex-16 questionnaire in patients with skin diseases in Morocco. J Dermatol Treat. 2014 Apr 1;25(2):106–9.

35. Odetunde MO, Odole AC, Odunaiya NA, Odetunde NA, Okoye EC, Mbada CE, et al. Cross-cultural adaptation and validation of the Igbo language version of the stroke-specific quality of life 2.0. Pan Afr Med J [Internet]. 2020 [cited 2025 Jan 29];37. Available from: https://www.panafrican-med-journal.com/content/article/37/111/full

36. Araya LT, Gebretekle GB, Gebremariam GT, Fenta TG. Reliability and validity of the Amharic version of European Organization for Research and Treatment of cervical Cancer module for the assessment of health related quality of life in women with cervical cancer in Addis Ababa, Ethiopia. Health Qual Life Outcomes. 2019 Dec;17(1):13.

37. El Alami Y, Essangri H, Majbar MA, Boutayeb S, Benamr S, El Malki HO, et al. Psychometric validation of the Moroccan version of the EORTC QLQ-C30 in colorectal Cancer patients: cross-sectional study and systematic literature review. BMC Cancer. 2021 Dec;21(1):99.

38. Gadisa DA, Gebremariam ET, Ali GY. Reliability and validity of Amharic version of EORTC QLQ-C30 and QLQ-BR23 modules for assessing health-related quality of life among breast cancer patients in Ethiopia. Health Qual Life Outcomes. 2019 Dec;17(1):182.

39. Osman SM, Khalifa N, Alhajj MN. Validation and comparison of the Arabic versions of GOHAI and OHIP-14 in patients with and without denture experience. BMC Oral Health. 2018 Dec;18(1):157.

40. Bowden A, Fox-Rushby, Julia, Nyandieka, Lilian Nyamusi. Methods for pre-testing and piloting survey questions: illustrations from the KENQOL survey of health-related quality of life. Health Policy Plan. 2002 Sep 1;17(3):322–30.
