# Supplementary material for: Measuring health-related quality of life in Africa: a systematic review of validated disease-specific and generic measurement tools
Source: Front Psychol. 2026 Jan 7;16:1667712. doi: 10.3389/fpsyg.2025.1667712 (PMC12819306; doi:10.3389/fpsyg.2025.1667712)
Supplement: Supplementary file 4 [file Data_Sheet_3.PDF]

Measuring health-related quality of life in Africa: a systematic review of validated disease-specific and generic measurement tools

Table 3a: Characteristics of first named authors of the included articles

Ref. No.	First Author	Gender	Primary Affiliation	Secondary Affiliation	Country of Primary Affiliation
1	Van Biljon(25)	NPA	Africa Unit for Transdisciplinary Health Research, Faculty of Health Sciences, North-West University, Potchefstroom, South Africa	None	South Africa
2	Smith(54)	Female	Department of Sport and Movement Studies, Faculty of Health Sciences, University of Johannesburg, South Africa	None	South Africa
3	Reba(22)	NPA	Department of Adult Health Nursing, School of Health Science, College of Medicine and Health Science, Bahir Dar University, P.O. Box 79, Ethiopia	None	Ethiopia
4	Westmoreland(55)	Female	Cancer Program, UNCProject-Malawi, Lilongwe, Malawi	Department of Paediatrics, Paediatric Haematology-Oncology, University of North Carolina, Chapel Hill, North Carolina	Malawi
5	Younsi (19)	Male	Department of Applied Research in Quality of Life, University of Sfax, Airport Road, km 1, LP 1099, 3003 Sfax, Tunisia	None	Tunisia
6	Ibrahim (20)	Male	Department of Physiotherapy, Faculty of Allied Health Sciences, College of Health Sciences, Bayero University Kano, Kano State, Nigeria	Department of Physiotherapy, Muhammad Abdullahi Wase Teaching Hospital, Hospital Management Board, Kano, Kano State, Nigeria	Nigeria
7	Jikamo (23)	Male	Department of Epidemiology and Biostatistics, Institute of Public Health, College of Medicine and Health Sciences, University of Gondar, Gondar, Ethiopia.	School of Public Health, College of Medicine and Health Sciences, Hawassa University, Hawassa, Ethiopia	Ethiopia
8	Mbada (26)	Male	Department of Medical Rehabilitation, College of Health Sciences, Obafemi Awolowo University, Ile – Ife, Nigeria	African Population and Health Research Centre, Nairobi, Kenya	Nigeria
9	Namisango (27)	Female	Departments of Clinical Epidemiology and Biostatistics, Makerere University, Kampala, Uganda		Uganda
10	Colbourn (24)	Male	Parent and Child Health Initiative (PACHI), Amina House (top floor, Entrance 3), Paul Kagame Road roundabout, P.O. Box 31686, Lilongwe 3, Malawi	UCL Centre for International Health and Development, 30 Guilford Street, London, WC1N 1EH, UK	Malawi
11	Guermazi (28)	Male	Department of Physical and Rehabilitation Medicine, Habib-Bourguiba Hospital, 3000 Sfax, Tunisia	Unite´ de recherche sur les pathologies de l'appareil locomoteur 04/UR/08-07, South University, Sfax, Tunisia	Tunisia
12	Mgbeojedo (56)	Female	University of Nigeria, Enugu Campus, Enugu State, Nigeria	Nnamdi Azikiwe University, Nnewi Campus, Anambra State, Nigeria	Nigeria
13	Muhye (29)	Male	School of Public Health, College of Medicine and Health Sciences, Bahir Dar University, Bahir Dar, Ethiopia	None	Ethiopia
14	Scott (57)	Female	Department of Health and Rehabilitation Sciences, Faculty of Health Sciences, University of Cape Town, Observatory 7925, Cape Town, South Africa	None	South Africa

NPA- Not Publicly Available

Table 2b: Characteristics of first named authors of the included articles

Ref. No.	First Author	Gender	Primary Affiliation	Secondary Affiliation	Country of Primary Affiliation
15	Ravens-Sieberer (30)	Female	Department of Psychosomatics in Children and Adolescents, Research Unit Child Public Health, University Medical Centre Hamburg-Eppendorf, Martinistr. 52, 20246 Hamburg, Germany	None	Germany
16	Ehab (31)	Female	*Clinical Pharmacy Department, Faculty of Pharmacy, Ahram Canadian University, Giza, Egypt*	*None*	*Egypt*
17	Duracinsky (32)	Male	AP-HP, Saint-Louis Hospital, Department of Clinical Research (Patient-Reported outcomes Unit), Paris, France	AP-HP, Bicetre Hospital, Internal Medicine & Infectious Disease Department, Le Kremlin-Bicetre, France	France
18	Gqada (33)	NPA	Department of Surgery, Faculty of Health Sciences, University of Cape Town, South Africa	Surgical Gastroenterology Unit, Groote Schuur Hospital, South Africa	South Africa
19	Onagbiye (34)	Sunday	Physical Activity, Sport and Recreation Research Focus Area, Faculty of Health Sciences, North-West University, South Africa	None	South Africa
20	Ohrnberger (21)	Male	School of Public Health, MRC Centre for Global Infectious Disease Analysis, Imperial College London, London, England	None	United Kingdom
21	Okello (49).	Male	Department of Internal Medicine, Mbarara University of Science and Technology, P. O Box 1410, Mbarara, Uganda	Department of Medicine, University of Virginia Health System, Charlottesville, Virginia, USA	Uganda
22	Uwizihiwe (35).	Male	Centre for Global Health, Department of Public Health, Aarhus University, Bartholins Alle 2, 8000 Aarhus C, Denmark	College of Medicine and Health Sciences, University of Rwanda, Kigali, Rwanda	Denmark
23	Owolabi (50)	Male	Department of Medicine, University College Hospital, PMB 5116, 200001, Ibadan, Nigeria	None	Nigeria
24	Kidayi (36)	Male	Faculty of Nursing, Kilimanjaro Christian Medical University College, Moshi 2240, Tanzania	None	Tanzania
25	Brandt (37)	Female	Department of Physiotherapy, University of the Free State, Bloemfontein, South Africa	None	South Africa
26	Kulich (51)	Male	AstraZeneca R&D, Medical Science, Mölndal, S-431 86, Sweden	None	Sweden
27	Borissov (58)	Male	Kings College, London, UK	None	United Kingdom
28	Kondo (38)	Male	Department of Family Medicine, Aga Khan University, Dar es Salaam, Tanzania.	None	Tanzania
29	El Fakir (39)	Female	Department of epidemiology and public health, Faculty of Medicine, University Sidi Mohammed Ben Abdellah, Fez, Morocco.	None	Morocco
30	Getu (52)	Male	The First Affiliated Hospital, Zhengzhou University, Zhengzhou 450052, Henan, China.	School of Nursing and Health, Zhengzhou University, Zhengzhou 450001, Henan, China	China

NPA- Not Publicly Available

Table 2c: Characteristics of first named authors of the included articles

Ref. No.	First Author	Gender	Primary Affiliation	Secondary Affiliation	Country of Primary Affiliation
31	Olasehinde (40)	Male	Department of Surgery, Obafemi Awolowo University, Ile-Ife, Nigeria	African Research Group for Oncology, Osun, Nigeria	Nigeria
32	Nkurunziza (41)	Male	College of Medicine and Health Sciences, University of Rwanda, Huye, Rwanda	None	Rwanda
33	Farid (42)	Female	Institute of Global Health and Human Ecology, American University in Cairo and Clinical Research Department, Children's Cancer Hospital Egypt (CCHE-57357), Cairo, Egypt.	None	Egypt
34	El Fakir l(43)	Female	Department of Epidemiology, Clinical Research and Community Health – Faculty of Medicine, Fez, Morocco.	None	Morocco
35	Odetunde et al(44)	Female	Department of Medical Rehabilitation, Obafemi Awolowo University, Ile-Ife, Nigeria.	None	Nigeria
36	Araya et al(45)	NPA	Social and Administrative Pharmacy Unit, Department of Pharmaceutics and Social Pharmacy, School of Pharmacy, College of Health Sciences, Addis Ababa University, P.O. Box: 1176, Addis Ababa, Ethiopia	None	Ethiopia
37	El Alami et al(46)	Male	The Surgical Department, National Institute of Oncology, Rabat, Morocco	Faculty Of Medicine of Rabat, Mohammed V University, Rabat, Morocco	Morocco
38	Gadisa et al(53)	Male	Lecturer of pharmacology at Pharmacy department, College of Medicine and Health Sciences, Ambo University, Ambo, Ethiopia	None	Ethiopia
39	Osman et al(47)	Female	Department of Oral Rehabilitation, Faculty of Dentistry, University of Khartoum, Khartoum, Sudan	None	Sudan
40	Bowden et al(68)	Male	Health Policy Unit, London School of Hygiene and Tropical Medicine, UK	None	United Kingdom

NPA- Not Publicly Available
